# Transmission of Chronic Wasting Disease in Wisconsin White-Tailed Deer: Implications for Disease Spread and Management

**DOI:** 10.1371/journal.pone.0091043

**Published:** 2014-03-21

**Authors:** Christopher S. Jennelle, Viviane Henaux, Gideon Wasserberg, Bala Thiagarajan, Robert E. Rolley, Michael D. Samuel

**Affiliations:** 1 Department of Forest and Wildlife Ecology, University of Wisconsin, Madison, Wisconsin, United States of America; 2 Biology Department, University of North Carolina, Greensboro, North Carolina, United States of America; 3 Wisconsin Department of Natural Resources, Madison, Wisconsin, United States of America; 4 U.S. Geological Survey, Wisconsin Cooperative Wildlife Research Unit, University of Wisconsin, Madison, Wisconsin, United States of America; East Carolina University School of Medicine, United States of America

## Abstract

Few studies have evaluated the rate of infection or mode of transmission for wildlife diseases, and the implications of alternative management strategies. We used hunter harvest data from 2002 to 2013 to investigate chronic wasting disease (CWD) infection rate and transmission modes, and address how alternative management approaches affect disease dynamics in a Wisconsin white-tailed deer population. Uncertainty regarding demographic impacts of CWD on cervid populations, human and domestic animal health concerns, and potential economic consequences underscore the need for strategies to control CWD distribution and prevalence. Using maximum-likelihood methods to evaluate alternative multi-state deterministic models of CWD transmission, harvest data strongly supports a frequency-dependent transmission structure with sex-specific infection rates that are two times higher in males than females. As transmissible spongiform encephalopathies are an important and difficult-to-study class of diseases with major economic and ecological implications, our work supports the hypothesis of frequency-dependent transmission in wild deer at a broad spatial scale and indicates that effective harvest management can be implemented to control CWD prevalence. Specifically, we show that harvest focused on the greater-affected sex (males) can result in stable population dynamics and control of CWD within the next 50 years, given the constraints of the model. We also provide a quantitative estimate of geographic disease spread in southern Wisconsin, validating qualitative assessments that CWD spreads relatively slowly. Given increased discovery and distribution of CWD throughout North America, insights from our study are valuable to management agencies and to the general public concerned about the impacts of CWD on white-tailed deer populations.

## Introduction

As in humans [1], chronic diseases constitute an important threat to wildlife because of the potential for demographic and evolutionary consequences [2] that negatively impact host populations. Detection and monitoring of these diseases can be difficult because prolonged epizootics can result in low, usually undetected, levels of infection, morbidity, or mortality [3]. Evaluating the incidence and spatial dynamics of chronic wildlife diseases requires long-term studies that may be difficult to conduct in natural populations due to financial and logistical constraints. These issues limit the ability of wildlife managers to understand and predict wildlife disease epizootics, assess their impacts on natural populations, and evaluate alternative control strategies. Because of such complexity, modeling disease dynamics may be the only practical way to quantify the spatial and temporal patterns of chronic diseases in wildlife, evaluate alternative transmission mechanisms, predict the spread of the infectious agents across the landscape, and identify viable management options.

Among transmissible spongiform encephalopathies (TSE), chronic wasting disease (CWD) is a fatal neurodegenerative disease of free-ranging and captive cervids. First recognized in captive mule deer (*Odocoileus hemionus*) in Colorado in the 1960s [4], CWD has subsequently been detected in wild and captive cervids of 21 states and two Canadian provinces. The uncertainty regarding long-term demographic impacts of CWD on cervid population health [5]–[7], possible human and domestic animal health concerns, and economic consequences of CWD have led management agencies to seek effective strategies to control CWD distribution and prevalence. In the absence of a treatment or vaccine for CWD, the main tool available for disease management in free-ranging populations is either selective harvest of infected individuals [8] or non-selective harvest of deer in known affected areas [9]. While selective harvest is impractical, the long-term efficacy of non-selective harvest is uncertain; only New York has officiated a CWD recovery phase after detecting two positive deer out of 32,000 sampled (NY Department of Environmental Conservation). Given a lack of clear understanding of CWD transmission dynamics in wild cervids, limited management tools, and finite financial resources, control or eradication of CWD is a tenuous and controversial undertaking.

An important tool in wildlife disease management is the mathematical model, which can be constructed to estimate important disease and population parameters, and predict the consequences of alternative management strategies [10]. A crucial issue in modeling host-pathogen dynamics is determining how infectious contact rate is affected by host density [6], [11], [12]. Two contrasting modes of transmission are typically considered: density-dependent transmission (DD) when infectious contacts increase monotonically with host density and frequency-dependent transmission (FD) when infectious contacts are independent of host density [11], [12]. In addition, a variety of intermediate forms may be modeled as non-linear functions [11], [13]. The theoretical implications of these contrasting forms of transmission on host population dynamics vary from stable host-pathogen coexistence for DD transmission to either host or pathogen extinction for FD transmission [14]; with an outcome that also depends on disease mitigating factors such as prophylactics or vaccines (see [15] for review). Consequently, effective management options depend on which transmission mode predominates [6], [11] and what additional tools (e.g., sterilization [16]) are available.

Transmission of CWD has been assumed to be FD in mule deer [5], [17], but little empirical research has been conducted and prior analysis of harvested Wisconsin white-tailed deer (*O. virginianus*) was inconclusive [18]. In Wisconsin, CWD was discovered in 2001 in three male white-tailed deer harvested in the south-central part of the state [19] from a core affected area (544 km^2^) of highest prevalence covering parts of Dane and Iowa counties, likely originating from a single introduction event followed by spatial spread [20]. In general, prevalence is higher in males and increases with age [21], [22]. For CWD, and other wildlife diseases, rates of geographic spread are unknown or very difficult to determine despite new techniques for determining the spatial distribution of diseases [20], [23], [24]. Although there is evidence that infection rate in this core area has increased [25], modeling of hunter harvest data to determine temporal and spatial trends has proved challenging [22], likely because of low prevalence and slow spatial spread. Owing to low temporal heterogeneity in age-specific prevalence data, earlier modeling efforts of the Wisconsin system suggested that CWD was introduced at least 2–3 decades before it was discovered [18].

The hypothesis of FD transmission in deer is largely based on the assumption that female matrilineal social structure and site-fidelity limits infectious contact between female social groups [5], [26], [27]; a finding corroborated by Grear *et al.* [28]. Schauber and Woolf [6] challenged this notion on the grounds of insufficient empirical support and encouraged modeling CWD under a broader transmission framework. High CWD prevalence in captive deer herds [9], [29], positive correlation between prevalence and deer abundance indices [20], and deer congregation on winter range, around bait-piles, or at mineral licks [30]–[32] suggest that DD transmission is also a feasible mechanism. Furthermore, behavioral and social differences between sexes or seasons (in both white-tailed and mule deer) may drive differences in CWD prevalence and transmission [7], [21], [22].

In this study, we investigated alternative modes of CWD transmission and evaluated the consequences of recreational harvest management on the dynamics and control of CWD in free-ranging white-tailed deer in south-central Wisconsin. Specifically, we built upon our previous work in this system [18] to test and compare seven transmission models accounting for DD, FD, and non-linear (NL) intermediate transmission functions. We also tested for sex-specific variation in infection rates, allowing for homogeneous or sex-specific model structure. We fit our matrix model to existing data in south-central Wisconsin using a maximum-likelihood approach to assess: (1) CWD infection rates with respect to host density vs. disease prevalence (i.e., transmission mode), and sex; (2) the time since CWD was introduced and rate of spatial spread in south-central Wisconsin, and (3) the implications of our results for recreational harvest strategies to manage the disease.

## Methods

### Ethics statement

As part of the requirement for mandatory registration of harvested deer in Wisconsin, regardless of whether the animal was obtained on public or private land, hunters were required to allow tissue collection by the Department of Natural Resources for CWD testing.

### Study area and data

The study area for our CWD analysis was focused on 544 km^2^ in south-central WI (core area of proposed CWD origin [20]), characterized by the highest prevalence within the CWD management zone (≈23,310 km^2^ [33]). We obtained data from the WI DNR in the south-central core area (hereafter core) of WI, using 15,136 records of hunter harvested white-tailed deer obtained between October and January 2002–2011 for parameter estimation, and 1,637 records between October and January 2011–2013 for validation of model predicted prevalence (Table S1). Of these samples, brain stem (obex) or retropharyngeal lymphatic tissue from 958 animals tested positive for CWD using immunohistochemistry or ELISA [34]. We classified each record by CWD status (positive/negative), sex, and five age groups; fawns, 1-year-olds, 2-year-olds, 3-year-olds, and >3 year-olds. Data from the core was used to evaluate the best supported transmission mode (next section). For analysis of spatial spread, we analyzed five surrounding regions, similar in size to the south-central core and each with 1,685 to 8,945 harvested deer, of which 18 to 298 tested CWD positive (with prevalence ranging from 2–4%). Although deer densities in the core area varied during the study, primarily in response to changes in harvest regulations and rates [33], there is no evidence that CWD-induced mortality was responsible for measureable variation in deer abundance.

### Model structure and selection

We used a multi-state non-spatial deterministic matrix model [18], [35], which accounts for age, sex, infection-stage, and seasonal (i.e., semi-annual time step: summer-winter) heterogeneity with respect to demographic, epidemiologic, and harvest parameters. Full details are provided in the Methods S1 section with a model structure diagram (Figs. S1 & S2) and component matrices (Figs. S3, S4, S5). As CWD infections are always fatal [9], we used a projection matrix with no recovery from the four disease stages in our model [35]–[37]. Fecundity and non-hunting survival rates were provided by the Wisconsin Department of Natural Resources (WDNR) (see Table S2) and following the detection of CWD in 2001, after which our prevalence data begins, we used estimates of sex-specific harvest provided by the WDNR.

Historical information about changes in deer density in the study area is subject to considerable uncertainty and our best estimate (given harvest records from Iowa County, WI, which is located in the study area) is that deer density was near zero in the early 1940s, but reached 9.3 deer km^−2^ just prior to CWD discovery in 2001. For simplicity, we simulated past deer population growth to achieve this density threshold using a logistic model (see Methods S1 for details).

We initiated population and disease dynamics based on a stable-age distribution obtained by using sustainable harvest rates of 48% and 26% for antlered and antlerless deer as per WDNR (see Methods S1). We assessed the potential sensitivity of model parameter estimates to the initial stable-age distribution by also using the sex-age structure from the harvest data to project the simulated deer population, but found negligible differences in model fit. For each model, we introduced CWD with one 2-year-old female into a simulated deer population that grew according to the demographic and harvest parameters available (see Methods S1). We simulated CWD potential introduction each year between 1945 and 2000 as historical records suggest that deer were effectively extirpated in southern Wisconsin prior to 1945. Based on previous simulations [18], models are not sensitive to the age or sex class initiating CWD in the population, however, increasing the initial number of infected deer results in lower time since disease introduction (*TDI*) estimates.

We evaluated seven alternative sex-specific transmission models and estimated infection coefficients (*β*s) and *TDI* under mixtures of density-dependent (DD) and frequency-dependent (FD) transmission. We also estimated infection coefficients for a non-linear (NL) model whose parameter values can specify a structure that is intermediate between DD and FD [11]. The mode of transmission or contact structure determines the formulation of the force-of-infection (*λ*, the instantaneous rate at which a susceptible acquires infection). Assuming homogeneous infectious contacts among and between each sex *i*: *λ_i_* = *β_i_*•*I* for DD-transmission [10] and *λ_i_* = *β′_i_*•(*I*/*N*) for FD-transmission [38], with infection coefficient *β_i_* or 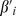, number of infected individuals (*I*), and total number of individuals (*N*). For our non-linear function, *λ_i_* = (*β_i_*•*I*)/(1−*ε_i_*+(*ε_i_*•*N*)) with scaling coefficient *ε*, which ranges from 0 to 1 [11]. As *ε*→0, *λ_i_* = *β_i_*•*I* and as *ε*→1, *λ_i_* = *β′_i_*•(*I*/*N*).

The form of our models do not control or specifically estimate directional transmission from the environment or model the dynamics of an environmental reservoir, thus our empirical infection rate estimates are likely a weighted combination of various direct and indirect transmission mechanisms which may depend on seasonal contacts among deer or with contaminated environments (see [39]). All age and sex groups are able to transmit and receive CWD, but for the most general model (i.e., with sex-specificity) the annual infection rate is constant across ages for each sex. For illustration, if we consider a WAIFW matrix (Who Acquires Infection From Whom; [10]) for male and female deer, where columns correspond to infectors and rows correspond to receivers of infection, our sex-specific transmission structure follows from:
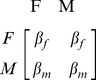
Thus, our models assume females receive infection from females and males at the same rate (via the *β*_f_ coefficient), while males receive infection from females and males with infection coefficient (*β*_m_). Infection rates are derived by the expression 1-exp(−*β_i_*•Δ*t*), where Δ*t* is equal to 0.5 year. In earlier modeling efforts, we attempted to estimate directional transmission coefficients (e.g., *β*_fm_ or the transmission coefficient for females receiving infection from males), but our data was not sufficient to support such complex model structures.

We used maximum-likelihood (*L*) profile analysis [40] with a binomial likelihood function based on annual CWD prevalence to estimate model parameters and compare relative fit (of model predicted prevalence) to observed prevalence data for hunter-harvested white-tailed deer from 2002 to 2010 in the CWD core area. The form of this likelihood function was:
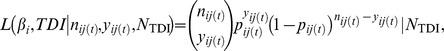
where *n_ij_*_(*t*)_ is the sample size of all hunter-harvested deer tested for CWD in year *t* (2002–2010), age class *j* (fawns, yearlings, 2, 3, and 4+ year olds), and sex *i*, *y_ij_*_(*t*)_ is the number of hunter-harvested deer that tested positive for CWD in year *t*, age class *j*, and sex *i*, and *p_ij_*_(*t*)_ is the model-predicted probability (given *β_i_* and *TDI*) that hunter-harvested deer in year *t*, age class *j*, and sex *i* were CWD positive. *N*_TDI_ is the simulated deer population vector distributed with stable age distribution in that year (*TDI*<2002), which corresponds with the estimated year of introduction of an index CWD infected (stage I) 2-year-old female. We evaluated goodness-of-fit of the most general model using Pearson's chi-squared test, and for each model, calculated Quasi-likelihood Akaike Information Criterion (QAIC) as −(2•ln(*L*)/*ĉ*)+2*n*, where *n* = number of parameters and *ĉ* is the variance inflation factor [41]. We made subsequent model comparisons using QAIC weights (*w*) [41] and used 2011 and 2012 harvest data (Oct 2011 to Jan 2013) to validate the predictive capability of our best supported model.

We note that deer density was not included in our Likelihood function; instead we used historic deer demographic, harvest, and density information to predict a plausible progression of deer density over time. Therefore, infection coefficients and *TDI* estimates were based solely on evaluation of observed and model predicted CWD prevalence (given the model simulated deer population) according to sex and age during the 2002 to 2010 harvest seasons. Optimally, it is preferable to incorporate both deer densities and prevalence into a Likelihood function for parameter estimation.

### Sensitivity analysis

We evaluated model sensitivity for predicted CWD prevalence and deer density 25 years after the last year of observed data (i.e., 2035) to variations in estimated model parameters *TDI*, *β*s, *γ* (probability of advancing to brain infection over a 6-month period; Fig. S1), fecundity, and harvest using Latin Hypercube sampling [42]. We used a semipartial correlation coefficient (*SPC*) to measure the correlation between each model parameter and output variable, corrected for other correlated parameters [43].

### Rate of spread

**Figure 1:**
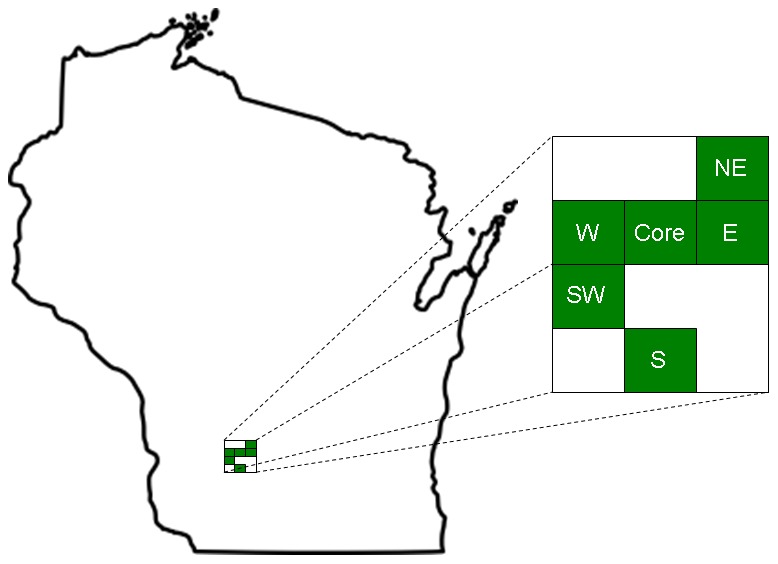
Map of study area including the southwestern core (544 km^2^ area of expected CWD origin in southwestern Wisconsin), and surrounding approximately core-sized regions used to estimate CWD spread.

To estimate the geographic rate of CWD spread across southern Wisconsin, we used the best supported transmission model from the core area (sex-specific FD model – see Results) to calculate sex-specific infection coefficients and *TDI* for five core-sized regions where data were sufficient to achieve model convergence (Fig. 1). We note that parameter convergence was not possible in other surrounding regions because of small sample sizes. Regions we considered ranged from ≈20–40 km from the center of the core area. We regressed the linear distance between the centers of the core and each region versus the respective difference in disease introduction time (*TDI*), thus the core area is represented by 0 distance and 0 Δ*TDI* relative to other areas. Available evidence suggests that the core contains the spatial introduction point of CWD in the study area [20], [22], [44], and no other published work suggests otherwise. Given this nexus for disease spread and initial arrival time, we used regression through the origin. The resulting slope of this distance-time relationship estimates the rate of CWD spread (km year^−1^) from the core area. Our calculation also assumes that average disease spread has occurred uniformly outward and likely represents the rate of spread early in the CWD epizootic. This assumption appears reasonable for locations near the core area [44].

### CWD dynamics and alternative harvest strategies

With infection coefficients from the best supported model (sex-specific FD model – see Results), we investigated the effect of three sex-specific harvest strategies on predicted CWD prevalence and deer density. These represent a range of possible management actions to address sex-specific FD CWD transmission using recreational harvest. The strategies included a *female-focused* harvest (approximately 50% female and 25% male harvest rates), a *herd-control* harvest based on average harvest rates since CWD discovery in Wisconsin in 2002 (27.7% females, 21% males), and a *male-focused* harvest (25% females, 50% males). To accommodate differences between agency harvest goals and realized harvest (*RH*) by hunters [45], we assumed density-dependent harvest in year *t*, strategy *s*, and sex *i* using a post-harvest societal tolerance level (*N*_tol_) in the core area of 5,040 deer. We subjectively designated this regulatory effect of societal tolerance for deer (*N*_tol_ = 9.3 deer km^−2^ or 9.3•544 km^−2^≈5040 deer) because prior to CWD discovery in the study area, harvest registration data suggested this asymptotic level of deer density was maintained in the study area. Thus, *RH_is_*_(*t*)_ = (*N_is_*_(*t*)_/*N*_tol_)•*h_is_*_(*t*)_, where *h_is_*_(*t*)_ is the nominal harvest rate with imposed constraints on *RH*, such that 10%≤*RH_is_*≤50%. All projections were applied to a modeled deer population beginning in 2011 and followed for ≈50 years.

We also projected CWD prevalence and deer density for a deer population where harvest is either precluded or substantially restricted such as in national parks, urban areas, or some private lands that restrict hunting. In this *no-harvest* scenario we imposed density-dependent fecundity (*f*_DD_) at a deer carrying capacity (*K*) of ≈77 deer km^−2^; the expected carrying capacity in south-central Wisconsin (WDNR). The functional form we used was *f*_DD(*t*)_ = *f*_(*t*)_−((*N*_(*t*)_/*K*)•*f*_(*t*)_), where (*N*_(*t*)_/*K*) is constrained to be ≤1, such that values of *f*_DD_ range from zero to values of nominal fecundity (*f*) at any time *t* (see Methods S1). We initiated dynamics with a post-harvest deer population of 9.3 deer km^−2^, assumed a stable age distribution, and introduced CWD into the simulated disease free population with a 2-year-old female. All matrix model calculations, likelihood calculations, sensitivity analysis, and projections were computed using MATLAB (Mathworks Inc., R2011a), while we used SAS v9.2 (SAS Institute Inc., Cary, NC, USA) for the rate of spread regression and diagnostics.

## Results

### Alternative transmission models

We found that sex-specific FD transmission was strongly supported by the data with *w* = 0.99 (next most parsimonious model had Δ*QAIC* of 10 units) (Table 1). Pure DD with equal sex infection coefficients was the least supported model with virtually no support from the data (Δ*QAIC* = 112), while our non-linear function also had negligible support (Δ*QAIC* = 35). The data were not sufficient to accommodate the sex-specific non-linear model. The infection coefficients (*β*s) for the best model (sex-specific FD) were 0.62 (95% CI: 0.56–0.67) for females and 1.20 (95% CI: 1.14–1.31) for males, with a *TDI* of 40 years (95% CI: 37–43) (Table 1).

**Table 1:** Alternative CWD transmission models used to estimate infection coefficients (*β*) and time since disease introduction (*TDI*) of chronic wasting disease in white-tailed deer from south-central Wisconsin during the 2002–2010 harvest seasons.

Model	*k* b	Δ*QAIC*	*TDI*	*β*	
				Male	Female
FD(F) FD(M)	3	0	40 (37–43)	1.20 (1.14–1.31)	0.62 (0.56–0.67)
FD(M) DD(F)	3	10	45 (44–55)	1.36 (1.31–1.43)	1.19×10^−4^ (1.08–1.27×10^−4^)
NL(F = M)^c^	2	35	34 (31–37)	0.83 (0.0122–0.93)	
FD(F = M)	2	36	34 (32–35)	0.90 (0.89–0.92)	
FD(F) DD(M)	3	38	42 (28–69)	2.08×10^−4^ (1.98–2.27×10^−4^)	0.88 (0.85–0.90)
DD(F) DD(M)	3	109	29 (27–31)	2.31×10^−4^ (2.15–2.50×10^−4^)	1.82×10^−4^ (1.71–1.91×10^−4^)
DD(F = M)	2	112	29 (28–30)	2.00×10^−4^ (1.99–2.00×10^−4^)	

a Akaike weight [41] for second best model was <0.01, while other models had support near zero.

b Number of model parameters.

c For the scaling coefficient *ε* of the non-linear model structure, the MLE was 0.94 with 95% CI of 0.23 to 1.0.

We used Quasi-likelihood Akaike Information Criterion (*QAIC*) for model comparison, with the best model having a *QAIC* of 223.11 and Akaike weight = 0.99^a^. These models evaluate transmission modes including density-dependent (DD), frequency-dependent (FD), and non-linear transmission (NL) as a function of sex specificity. Estimated parameters include 95% confidence intervals.

**Figure 2:**
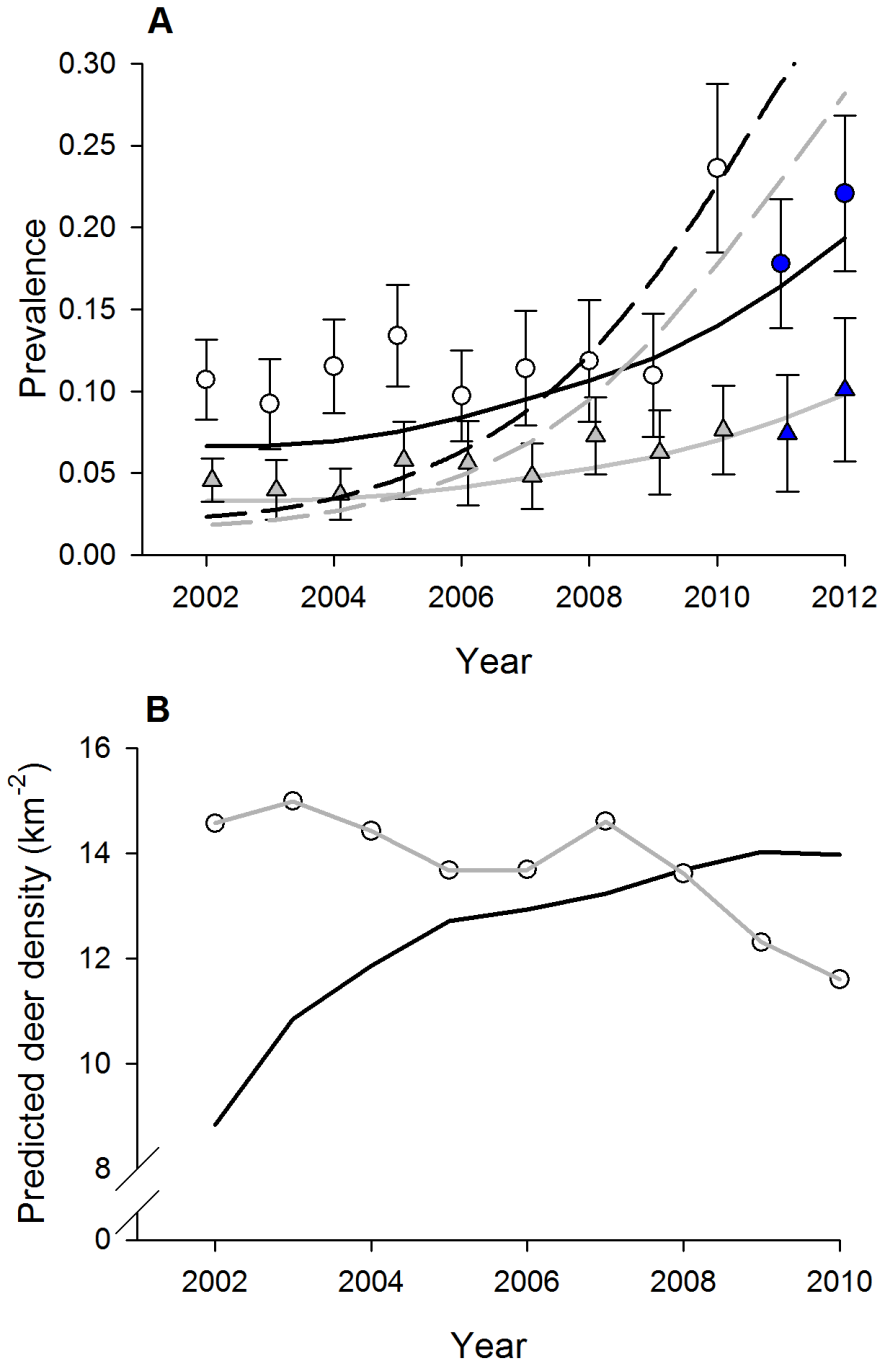
A) Observed female (grey ▵) and male (○) prevalence (95% CI) using data from the south-central core area (544 km^2^) of Wisconsin from the 2002–2010 harvest seasons including sex-specific model predictions under frequency- (FD) or density-dependent (DD) transmission: FD female (solid grey), FD male (solid black), DD female (dashed grey), and DD male (dashed black). **B) Predicted deer density of females (grey) and males (black) over the years of observed data using observed harvest rates.** Note that observed data for the 2011–2012 harvest seasons (blue-filled icons) in panel A were not used in parameter estimation, and are presented here to support validation of the predicted model.

For the sex-specific FD model, observed and model-predicted prevalence resulted in good fit for females (*χ^2^* = 47.73, *df* = 52, *P* = 0.64), but poor fit for males (*χ^2^* = 204.44, *df* = 52, *P*<0.001) where there was significant divergence between observed and predicted prevalence for several years (Fig. 2A). Predicted deer density using observed harvest rates over the years of observed data differed less than 5% for the sex-specific FD and DD models, so we only show densities for the FD model (Fig. 2B). Using the goodness-of-fit statistics, we estimated a variance-inflation-factor (*ĉ*) of 2.43, indicating mild overdispersion likely due to spatial and/or temporal dependence of CWD prevalence in the study area. The estimated variance of infection coefficients and TDI parameters (*θ*) were inflated accordingly by *ĉ* • var(*θ*). Although this adjustment may account for all or part of the overdispersion in the data, precision of the model parameters may still be overestimated. We validated our sex-specific FD model predictions with data from 2011 and 2012, which resulted in good fit for adult females (*χ^2^* = 5.32, *df* = 5, *P* = 0.38) and adult males (*χ^2^* = 9.32, *df* = 5, *P* = 0.10).

We evaluated temporal autocorrelation of predicted prevalence by examining the 1^st^ order autocorrelation of the residuals from the FD-sex model using a Durbin-Watson test for males and females separately. For females the test indicated positive 1^st^ order autocorrelation with residuals (*d* = 0.851, *df* = 1, *P* = 0.03), while for males 1^st^ order temporal autocorrelation in residuals was not detected (*d* = 2.19, *df* = 1, P = 0.10).

### Sensitivity analysis

The sensitivity analysis conducted on the sex-specific FD model showed that CWD prevalence in 25 years was negatively correlated to the harvest rate of antlered deer (*SPC* = −0.64) and tended to increase with harvest of antlerless deer (*SPC* = 0.41) (Table 2). Antlerless harvest had the most impact on deer density in 25 years, with abundance declining for increasing harvest of adult does and fawns (*SPC* = −0.80; Table 2). We found at best a weak influence of parameters *β*, *γ*, and *TDI* on future prevalence and deer density. We evaluated a range of starting deer population sizes in the core area for our simulations, ranging from 2 through 544 (≤1 deer km^−2^). We found that starting population size did not affect the ability of the models to fit the data, and infection coefficient estimates and *TDI* varied <5% across different starting population sizes.

**Table 2:** Sensitivity of primary model parameters on predicted prevalence of CWD infection and deer density after 25 years (2035).

Input parameters	Distribution^a^	Prevalence^b^	Deer density^b^
*TDI*		0.09±0.08 (0.72)	−0.02±0.04 (0.92)
*β*_male_	N({40,1.20,0.62},∑)^c^	0.20±0.09 (0.40)	−0.05±0.04 (0.82)
*β*_female_		0.22±0.10 (0.34)	−0.07±0.04 (0.78)
*γ*	N(0.5,0.08^2^)^d^	−0.01±0.08 (0.96)	0.01±0.05 (0.98)
Harvest antlered deer	N(0.5,0.009)^e^	**−0.64±0.10** (0.003)	0.15±0.05 (0.53)
Harvest antlerless deer	N(0.25,0.01)^e^	0.41±0.11 (0.07)	**−0.80±0.08** (<0.001)

a We used Latin Hypercube Sampling for each parameter with *S* = 20 equal probability intervals and one random value from each interval. Values for each parameter were paired randomly with values from all other parameters.

b Mean *SPC* ± *SE* between input parameters and prevalence or deer density in 2035; stochastic analysis based on *S* = 20 replications. Probability of a *t*-statistic (with *S*-2 df; [43]) that evaluates *SPC* = 0 provided in parentheses.

c Because *β*_male_, *β*_female_, and time since disease introduction (*TDI*) are correlated, we used a trivariate normal distribution, N({*TDI*,*β*_male_,*β*_female_}, ∑), where ∑ = variance-covariance matrix, calculated for parameter combinations within the 95% confidence region [69]; *TDI* rounded to closest integer.

d Transition (γ) from lymph-node positive (I) to obex-positive (O) represents differences in CWD progression among deer genotypes in terms of CWD susceptibility; standard error (*SE*) derived from 95% CI bounds = 8–16 months (e.g., representing transition to Obex infection for the two common genotypes).

e Gaussian distributions for harvest rates of antlered and antlerless deer are based on mean hunting rates and coefficients of variation (0.18 and 0.15, respectively) during 2002–2010 in the core area.

We used Latin Hypercube Sampling and a semi-partial correlation coefficient (*SPC*) to measure the relative influence of model parameters; significant coefficients are bolded.

### Rate of spread

**Figure 3:**
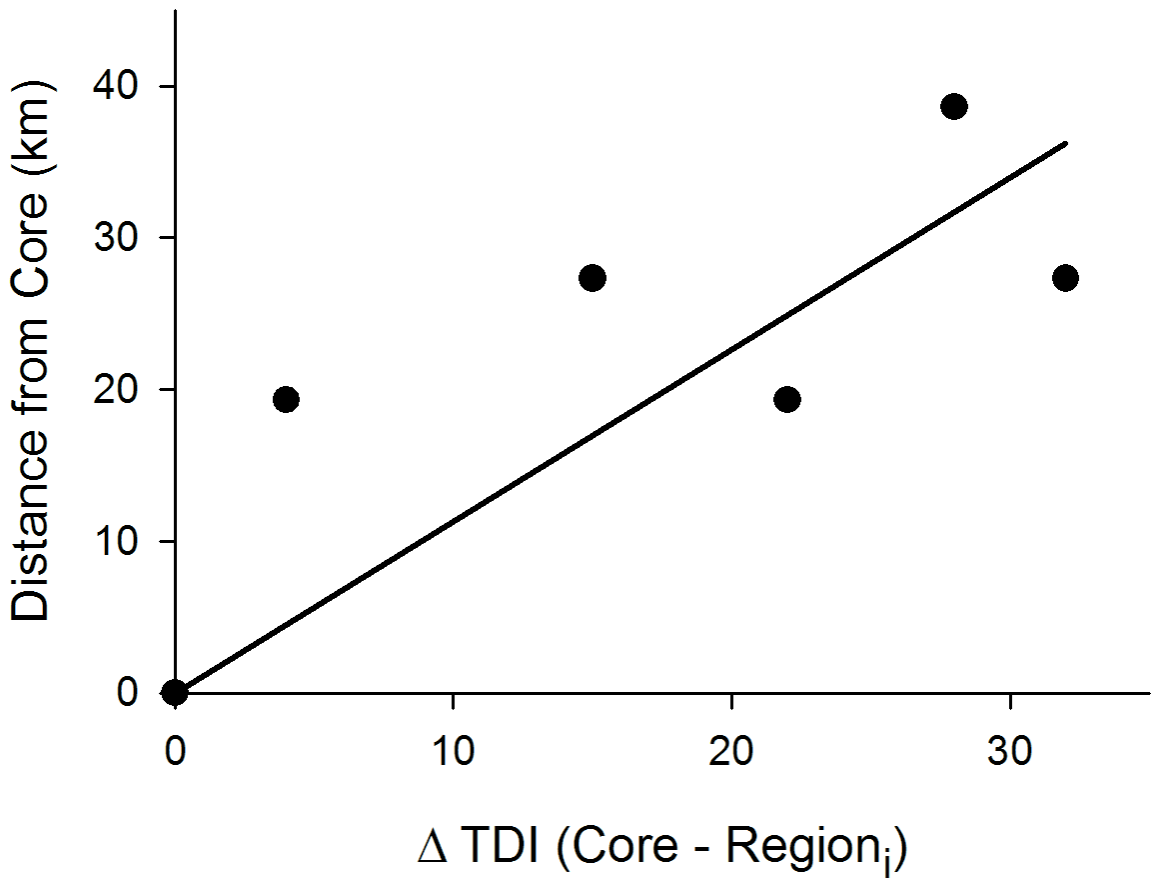
Plot of points for zero-intercept linear regression of distance from the core versus difference in TDI (Core – Region*_i_*) in years. The estimated slope with adjusted *R^2^* = 0.87 (*F*_1,4_ = 26.82, *P* = 0.007) was 1.13 (*SE* = 0.22), suggesting CWD spread across the south-central core area of Wisconsin was on average approximately 1.13 km yr^−1^.

For areas west and southwest of the core, infection coefficients were significantly greater than in the core for male deer (Table 3). For regions outside the core, *TDI* estimates were significantly lower indicating later CWD establishment, with the exception of an area east of the core where sparse data resulted in an unidentifiable upper confidence bound (Table 3). A zero-intercept linear regression of distance from the core versus difference in *TDI* had a significant adjusted *R^2^* = 0.87 (*F*_1,4_ = 26.82, *P* = 0.007) with slope parameter of 1.13 (*SE* = 0.22), indicating an average geographic rate of spread of CWD in the vicinity of the western core of south-central Wisconsin of 1.13 km year^−1^ (Fig. 3).

**Table 3:** Maximum-likelihood estimates (and 95% confidence intervals) for infection coefficients (*β*) and time since disease introduction (*TDI*) of chronic wasting disease in white-tailed deer in core-sized regions (≈544 km^2^) surrounding the south-central core in Wisconsin from 2002–2010.

Direction (distance)^a^	*β* Male	*β* Female	*TDI*
North-East (27 km)^b^	1.29 (0.70–2.05) *P* = 0.795	1.08 (0.66–1.70) *P* = 0.085	**8 (3–32) ** ***P*** **<0.001**
East (19 km)^c^	1.49 (1.08–1.91) *P* = 0.192	0.48 (0.24–0.74) *P* = 0.307	36 (23-NA)^d^
South (39 km)^b^	1.58 (1.11–2.16) *P* = 0.162	0.79 (0.46–1.19) *P* = 0.368	**12 (5–32) ** ***P*** **<0.001**
South-West (27 km)^c^	**1.64 (1.28–2.04) ** ***P*** ** = 0.033**	0.57 (0.35–0.81) *P* = 0.691	**25 (10–38) ** ***P*** ** = 0.042**
West (19 km)^c^	**1.55 (1.31–1.78) ** ***P*** ** = 0.011**	0.77 (0.61–0.91) *P* = 0.087	**19 (13–29) ** ***P*** **<0.001**

a Direction and distance from the center of the core to the center of a given region.

b no *ĉ* correction; North-East: *χ^2^* = 5.64, *df* = 8, *P* = 0.69; South: *χ^2^* = 7.07, *df* = 13, *P* = 0.90.

c *ĉ* correction; East: *χ^2^* = 22.53 *df* = 14, *P* = 0.07, *ĉ* = 1.61; South-West: *χ^2^* = 29.63, *df* = 16, *P* = 0.02, *ĉ* = 1.85; West: *χ^2^* = 27.85, *df* = 16, *P* = 0.03, *ĉ* = 1.74.

d The 95% CI upper bound was not estimable.

The transmission model assumes sex-specific FD transmission. *P* are the z-test probabilities evaluating the null hypothesis that parameter values are equal between the core and a given region; bold values indicate *α*≤0.05.

### Harvest strategies

**Figure 4:**
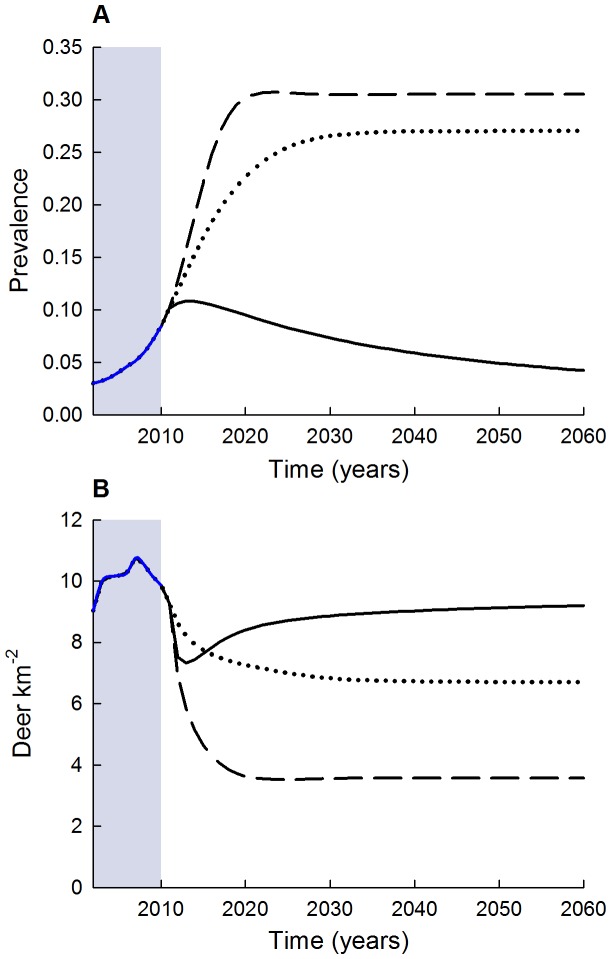
Predicted CWD population prevalence (A) and deer density (B) using transmission estimates from the best supported sex-specific frequency-dependent model. Three strategies were considered including male-focused harvest rates (solid line; female = 25%, male = 50%), herd-control harvest (dotted line; female = 28%, male = 22%), and female-focused harvest (dashed line; female = 50%, male = 25%). Note that the herd-control harvest strategy represents an average of the existing harvest conditions in the south-central core of WI during the 2002–2010 harvest seasons (blue shaded area).

**Figure 5:**
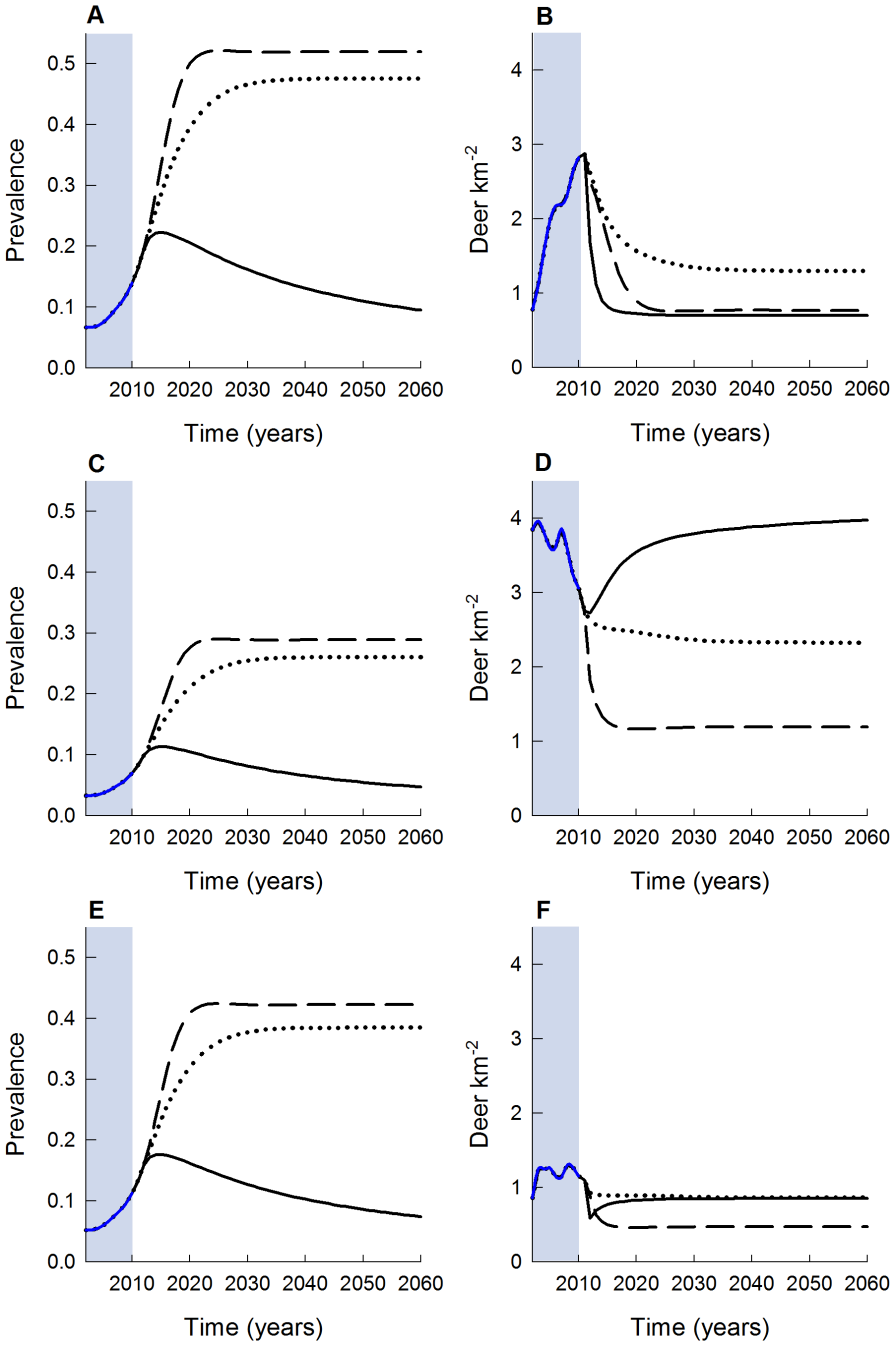
Predicted CWD prevalence (A, C, E) and respective deer density (B, D, F) for three harvest strategies using transmission estimates from the best supported sex-specific frequency-dependent model: male-focused harvest (solid line; female = 25%, male = 50%), herd-control harvest (dotted line; female = 28%, male = 22%), and female-focused harvest (dashed line; female = 50%, male = 25%). Panels A and B show adult males, panels C and D show adult females, and panels E and F show yearling males. Note that the herd-control harvest strategy represents an average of the existing harvest conditions in the south-central core of WI during the 2002–2010 harvest seasons. The areas shaded in blue represent the observed data years, and FD-sex model predictions are based on observed harvest rates.

**Figure 6:**
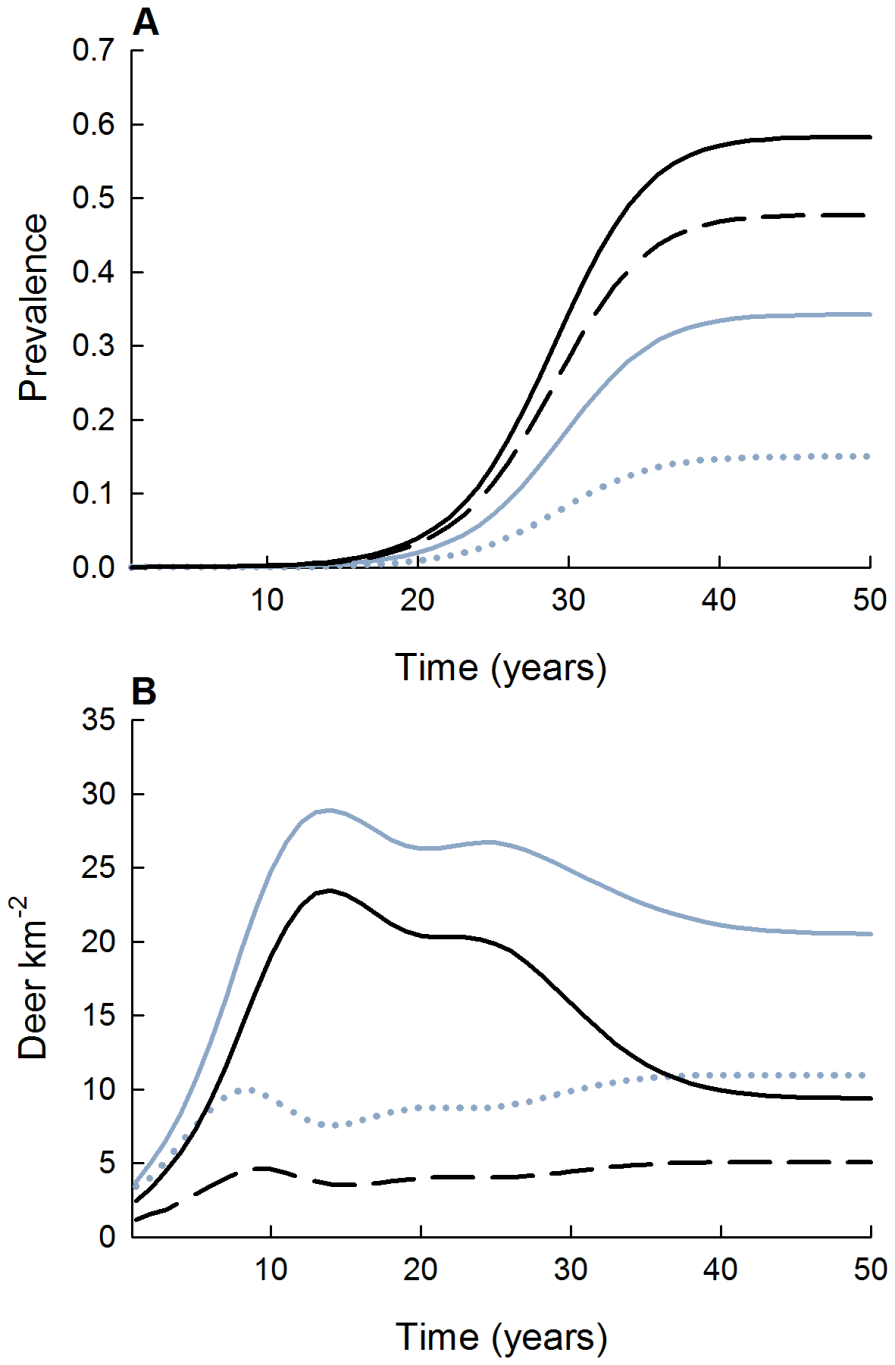
Predicted CWD prevalence (A) and deer density (B) for fawns (dotted), male yearlings (dashed), female adults (solid blue), and male adults (solid black) using transmission estimates from the best supported sex-specific frequency-dependent model. This scenario represents a no-harvest strategy, initiating CWD in a deer population with initial density of ≈9 deer km^−2^ with density-dependent fecundity as a population regulation mechanism (*K*≈77 deer km^−2^).

Of the three harvest strategies, male-focused harvest resulted in eventual decline of CWD prevalence to under 5% by 2060 (and 2.5% by 2110; not shown) and stable post-harvest deer density of ≈9 deer km^−2^ after 20 years (Fig. 4), resulting in a female-dominated population compared to other strategies (Fig. 5). Average realized harvest rates under this scenario were close to nominal rates at 24% and 49% for females and males, respectively. For both the herd-control and female-focused harvest strategies, projected CWD prevalence increased to 26% and 30%, while total post-harvest deer densities stabilized at ≈7 and 4 deer km^−2^, respectively (Fig. 4). The reduction in deer density resulted in average harvest rates that were considerably lower than nominal levels for females and males, respectively, with realized harvest rates of 20% and 16% for the herd-control, and 20% and 10% for the female-focused strategies. For a naïve population with no hunting, density-dependent fecundity as a regulatory population mechanism, and one introduced infectious individual, prevalence increased to 57% for adult males after 40 years (Fig. 6). Total deer density reached an asymptote of ≈46 deer km^−2^ after 40 years, although adults declined by as much as 50% during the near-exponential phase of CWD prevalence increase (years 25–40) (Fig. 6).

## Discussion

### CWD Transmission

Using white-tailed deer harvest data from south-central Wisconsin, we show that FD CWD transmission is the best supported model for both sexes (with higher infection rates for males) at a broad spatial scale, whereas our earlier efforts to model this system could not discriminate between FD and DD transmission [18]. It has been suspected that FD was a dominant transmission mechanism in mule deer [5], [17]; [ but see 6], and more recently in white-tailed deer [28], [46]. Furthermore, our modeling results suggest a more recent and biologically plausible time since CWD introduction in south-central Wisconsin compared with earlier analysis [18]. As demonstrated in previous work in this CWD system [21], [22], [25] and in Colorado [7], [47], adult males have higher CWD infection rates than females. Although the mechanism for higher CWD infection and prevalence in males is unknown, these differences may be driven by sex-specific social behavior [7], [21]. Males typically have larger home ranges, longer dispersal distances, interactions with other males, or rut-related behavior [48] that could result in more contacts with infectious deer. In contrast, females generally interact within a much smaller matrilineal group [27], [28], [49], and only briefly with males during rut [47].

Given the simplicity of our model, our estimated infection coefficients are likely a function of several different (and largely unknown) mechanisms that may vary between/among sexes, seasons, and the environment. These infection rates represent a weighted average of many potential drivers as summarized by Potapov et al. [39]. Our models do not account explicitly for indirect transmission from the environment where prions can persist for years [50], [51], although our infection rates implicitly subsume both direct and indirect routes of infection. The importance of environmental transmission has been demonstrated in captive mule deer [52], [53] and theoretical modeling indicates that population impacts can be driven by the length of time that prions remain infectious in the environment [54]. In the long term, the potential accumulation of an environmental reservoir of infectious prions may become an increasingly important component of CWD transmission; however, the relative contribution of direct and indirect transmission in wild deer populations remains unknown and requires further research. Although we expect infectious contact likely varies by sex and season, harvest data were insufficient to account for intra-annual complexity in sex-specific transmission. Additional insights for CWD management given directional sex-specific transmission (i.e., female-to-male, female-to-female) may require focal research studies that determine differences in infectious contact between and among sexes [49], [55]–[57] and how these influence the risk of disease transmission [28]. In particular, understanding the mechanisms that lead to rates of male infection twice as high as females could provide crucial insights on management strategies designed to reduce male CWD prevalence as an alternative to high male harvest.

Assuming CWD originated in the core area and environmental accumulation of prions contributes significantly to transmission, we would expect higher infection rate estimates in the core compared with surrounding areas. We are uncertain why infection rate is apparently greater in males for some areas to the west and southwest of the core. These surrounding areas have similar habitat characteristics with the core, and we would not expect deer abundance to vary significantly prior to CWD discovery. Heterogeneous harvest management conducted among areas may be one potential explanation. However, this difference also suggests that unidentified environmental characteristics or management actions may influence the current and future trends in CWD prevalence. Regardless, these patterns suggest that our model predictions for the core area may underestimate the rate of CWD increase in other areas. Future research is needed to understand the drivers of CWD transmission, how these vary spatially, and their influence on future patterns of infection. The identification of potential environmental reservoirs (e.g., common feeding areas, mineral licks) and evaluation of the significance of indirect transmission in free-ranging deer populations would also enhance our ability to predict future trends in infection and allow a better evaluation of alternative control strategies.

In concept QAIC should help account for overdispersion in our data, which might result from missing covariates in the model and/or a lack of independence in the data (e.g., [58]). Such lack of independence may be due to spatial and/or temporal autocorrelation, and while we do not explicitly account for such effects, we rely on QAIC to generally accommodate a portion of these impacts. While we detected significant temporal autocorrelation in residuals for predicted female prevalence, other research in the same study area [44], [46] found no spatial autocorrelation in model residuals using a 93.6 km^2^ or 2.6 km^2^ spatial frame, respectively. We caution that despite use of QAIC, our model parameter estimates may still be overly precise.

### Rate of spread

Several studies indicate that the southwestern core area of WI is the likely point of origin for CWD in our study area, with an inverse relationship between distance-to-core and prevalence as would be expected from an introduced disease spreading across the landscape [20], [22], [44]. To our knowledge, we present the first empirical estimate of CWD geographic spread, based on sex-specific FD transmission, which indicated a low average rate (1.13 km year^−1^) during initial phases of the epizootic. There is no current evidence to suggest that CWD spread in our study area was facilitated by humans (via movements of infectious animals between game farms or preserves); however, the anecdotal evidence of such events warrants further investigation. Though DD transmission was not supported by our data, the estimated rate of geographic spread was similar for this model structure. Our results suggest that in the south-central Wisconsin endemic area, CWD has slowly moved across the landscape and is probably not a recent development. Clearly this estimated rate of spread must be considered unique to the outbreak in south-central Wisconsin.

Rates of CWD spread in other regions are likely influenced by a number of factors including habitat features [44], [59], mode of disease transmission, host species (e.g., white-tailed or mule deer), population structure, host movements [60], dispersal [61], and possibly the environment [54]. For example, recent analyses [44] indicate that CWD may be spreading faster from the outbreak in eastern Wisconsin and northern Illinois than from south-central Wisconsin. Our simple estimate also assumes an average uniform diffusion from the point of origin and ignores potential disease movement via longer distance dispersal [60], although recent discovery of CWD in north-west Wisconsin does not appear to be linked to long-distance dispersal from southern Wisconsin based on genetic analysis (S. Robinson Pers. Comm.). In addition, our analysis does not account for habitat heterogeneity and physical barriers (natural or anthropogenic) that influence landscape scale movement and interaction of deer populations [62], [63] or CWD distribution [44], [63]. We also note that despite a highly significant R^2^ value, our simple regression utilizes only six data points (including the core, which we assume is the origin of the epizootic), with uncertainty that is not accounted for in the regression. As such, there is likely higher variance associated with our estimated rate of spread.

Despite these limitations, our estimate provides a starting place to conceptualize early CWD spread across the southern Wisconsin landscape. In the context of CWD, we believe the areas surrounding the core are currently in relatively early stages of the epizootic with low, but increasing prevalence. Under FD transmission and barring effective management efforts, CWD prevalence is predicted to increase over time, and we suspect that the rate of spread may also increase because more young males will become infected prior to dispersal [46]. As such, we consider our spread estimate as a lower bound that is likely to increase as the epizootic progresses.

### Harvest strategies

As a consequence of FD transmission, our simulations predict that in the next decade CWD prevalence can increase to relatively high levels (25% in females and 50% in males) in the absence of significant management actions to reduce infection rates. Of the three harvest strategies we evaluated, only male-focused harvest succeeded in reducing CWD prevalence below current levels. Prevalence is reduced because this strategy removes animals from the highest prevalence class (reducing infection rates), while allowing dilution of population-level CWD prevalence by recruitment of more females [64]. In contrast, CWD increased under female-focused and herd-control harvest strategies. By focusing harvest on the portion of the population with highest prevalence and infection rates, our simulation suggests that harvest management can effectively reduce prevalence despite FD disease transmission. Although disease eradication may not be possible, prevalence reduction (especially in higher risk groups), which reduces force of infection, is the key to mediating disease impacts on host populations in the long term. Effective disease management by sex-specific differential harvest has also been explored for bovine tuberculosis in deer [55].

The density-dependent harvest structure we imposed produced much lower average realized harvest (RH) rates for the female-focused and herd-control strategies, compared with male-focused harvest. High female harvest reduces population size, which requires lower realized harvest rates to maintain stable population goals (based on societal tolerance for deer). While this density-dependent harvest structure is artificial, it is intended to represent hunter effort in response to perceived deer densities. In the absence of such a mechanism, static harvest rates over the simulated time frame of 50 years resulted in host and disease extinction, as predicted in theoretical models of FD disease transmission [14]. In addition, our results show that deer demography and CWD dynamics are sensitive to changes in harvest. Estimation of unbiased harvest rates requires accurate information on both the distribution of harvested animals and the distribution of the underlying population. Although harvest-based estimates for deer populations have various limitations [9], [65], the importance of this parameter for monitoring the performance of CWD management programs suggests future research to improve estimation procedures should be considered.

The demographic implications of alternative harvest strategies for disease management are also important as they affect deer densities, recreational opportunities (e.g., hunting or observation), and potential disease spread. While male-focused harvest reduces CWD prevalence in the long term, it results in lower densities of adult males (compared with herd-control), which are usually of primary interest to deer hunters. For the herd-control harvest strategy (current deer management goals) nearly 50% of adult males and 25% of adult females are expected to become infected within another decade. Even worse, for female-focused harvest not only are deer densities expected to be low, but more than 50% of surviving adult males and 30% of adult females would be infected. In general, these harvest strategies are characterized by accelerating rates of infection in all deer, and higher prevalence, particularly in males. Considering the constraints of our model the tradeoff between strategies is clear; CWD can eventually be reduced with fewer opportunities to harvest healthy adult bucks, or more adult bucks may be available for harvest, but with higher rates of CWD infection. Given that quality deer management practices focus on production of older bucks with large antlers, management agencies could face difficult alternatives from these competing interests. However, if an efficacious CWD vaccine was available and cost-effectively distributed to broad segments of a deer population (particularly males), managers would have more flexibility to employ a disease control strategy combining harvest and vaccination to provide adequate recreational opportunities to harvest CWD-free deer.

The mechanism for density-dependent population regulation in deer is not well known, but one hypothesis is that deer reduce body size and maintain survival rates while lowering reproduction [66]. Therefore, we used density-dependent fecundity to regulate population size in our *no-harvest* simulations. The goal of these simulations was to illustrate the rapid increase in CWD prevalence and eventual impact on deer populations in the absence of harvest or other factors that remove infected animals prior to mortality from CWD. Such situations might be likely in high density urban deer populations, national parks, captive deer farms, or other areas where deer harvest or removal is limited. This simulation is not designed to represent current conditions in Wisconsin, and we consider this a worst-case disease scenario in areas without harvest. However, we also note that CWD transmission rates and prevalence are much higher in captive deer farms than has been reported in wild populations [67].

### Caveats

We highlight that our models do not specifically account for potentially important mechanisms such as environmental transmission, differences in Prnp genotypes [2] or infectious contact within matrilineal social groups [27], [28], which could contribute to future infection rates, and affect future predictions of CWD dynamics. For instance, infectious prions may accumulate in the environment causing increased future rates of environmental transmission. Our projections of CWD dynamics are limited because we cannot account for these unknown, but potentially important, effects on transmission due to accumulation of infectious prions in the environment over time. Two recent evaluations of the potential effects of soil characteristics (specifically clay content) on CWD transmission to yearling deer and on spatial patterns of CWD prevalence in Wisconsin [44], [46] failed to show an association between soil characteristics and CWD, unlike a similar study from Colorado [68]. While there is no current evidence supporting a significant role for environmental CWD transmission in Wisconsin, we cannot discount the possible influence this may have on future CWD dynamics in our study system. The relative importance of environmental and direct transmission is critical to understanding future CWD dynamics in wild deer.

Unfortunately, our data was collected in one season (winter) each year, making it impossible to estimate seasonal, environmental, or between/among sex infection rates without assumptions about the infectious contact structure between/among males and females, and with the environment. Spatial heterogeneity, deer aggregation, and the broad spatial scale of our study area could impact our estimated CWD transmission mode and infection rates. Although DD transmission could operate at finer spatial scales, a recent study of transmission in yearlings at a 2.6 km^2^ scale also indicates transmission is primarily FD [46]. Given the sparse data available at a fine scale, such analysis within our modeling framework was not possible. The purpose of this paper was not to fully describe the many different potential transmission mechanisms on CWD dynamics (which is a very desirable, but challenging goal). Rather, our goal was to evaluate relatively broad-scale dynamics of the disease and implications for disease management, given the available data for harvested deer in Wisconsin.

## Conclusions

Given our model structure and data, our results provide strong support for FD transmission of CWD with the force of infection driven by changes in prevalence, which we suggest is a vital metric for focused control efforts. Generally as prevalence increases, as found in Wisconsin, infection rate also increases in the absence of intervention, producing an accelerating pattern of infection. Assuming that frequency-dependent transmission predominates (as our evaluation indicates), management to reduce prevalence will mediate potential CWD population impacts. The higher rate of infection and prevalence in males, thus, provides the basis for effective CWD management using deer harvest focused on this sex. Management to reduce prevalence might be accomplished through the synergistic effects of targeted harvest and vaccination of males. Unfortunately, we know little about the mechanisms for male infection and further research is needed before alternative management strategies to reduce male infection rates can be developed. Spatial differences in CWD infection rates, despite similar habitat and pre-CWD deer abundance, suggest that unidentified environmental or management factors may also influence disease dynamics and future trends in prevalence. Future research to understand the drivers of CWD transmission, how these vary spatially, and the relative importance of environmental and direct transmission is critical to understanding future CWD dynamics in wild deer.

Our results also indicate that even with high deer densities CWD has been spreading at a relatively slow rate across the landscape; in agreement with larger scale spatial patterns for prevalence [44]. However, as disease prevalence continues to increase, the rate of infection in yearling bucks will also increase [46]. Because dispersing bucks may be an important source of disease spread, these patterns suggest that CWD prevalence outside the core area will continue to grow and the disease may spread at an increasing rate. Although the drivers of CWD spatial spread are not generally known (see [44] for identification of landscape features that affect spread), management efforts to reduce both local prevalence and deer abundance will likely reduce dispersal of infected yearling bucks. However, the relative impact of reducing deer abundance versus prevalence in lowering the number of infected yearling bucks likely depends on disease prevalence and deer density [46]. Further research is needed to determine the factors that affect spatial spread and develop effective management strategies.

## Supporting Information

Figure S1 Compartmental model structure of the CWD study system. CWD stages are based on disease progression using 0.5 year time-steps. All individuals are assumed to be born susceptible (S). Infection is first detectable in retropharyngeal lymph-nodes when animals are assumed to be infectious (I). The sex-specific S-to-I transition probability is *π_i_*. Infection of the brain stem is the next detectable infection stage (O) and usually takes up to 6 months after prion detection in the alimentary lymph-nodes. The I-to-O transition probability (γ) is, hence, assumed to be equal to one. The final stage of infection is brain vacuolization which occurs 10–12 months after initial brain infection and is commonly associated with clinical signs (C). Accordingly, the O-to-C transition probability (φ) is assumed to be 0.5. From this stage most animals die within 6 months. Therefore, the disease-induced mortality probability (α) is assumed to equal one. Deer survive within and between compartments with age-sex survival probabilities *s_ij_* for the *j* ^th^ age of sex *i* and reproduce with age-specific fecundity probabilities *f_j_*.
(TIF)

Figure S2 General matrix structure organization. The general hierarchical structure of the model where age-specific sub-matrices (20 6-month steps) of demographic, epidemiologic, and harvest parameters are nested within matrices accounting for four disease stages and both sexes. These matrices are further nested within season (summer and winter). Disease stages include S (Susceptible), I (Infectious), O (Obex brain positive), and C (Clinical) with F and M representing female and male deer, respectively.
(TIF)

Figure S3 Seasonal demographic matrices. Each seasonal demographic matrix accounts for four disease stages for each sex, and is composed of eight sub-matrix elements. Panels A and B contain the summer **D^(s)^** and winter **D^(w)^** demographic matrices, respectively and are composed of the following sub-matrix elements: sex-specific (indexed f or m) transition from Susceptible to Infectious stages (**Π_i_**), transition from Infectious to Obex positive stages (**Γ**), transition from Obex positive to Clincial stages (**Φ**), transition from Clinical stage to death (**A**), sex-specific survival (**S**), fecundity (**F**), identity (**I**), and zero sub-matrices (**0**).
(TIF)

Figure S4 Seasonal epidemiological matrices. Each seasonal epidemiological matrix accounts for four disease stages for each sex and is composed of five sub-matrix elements. Panels A and B contain the summer **E^(s)^** and winter **E^(w)^** epidemiological matrices, respectively and are composed of the following sub-matrix elements: sex-specific (indexed f or m) transition from Susceptible to Infectious stages (**Π_i_**), transition from Infectious to Obex positive stages (**Γ**), transition from Obex positive to Clincial stages (**Φ**), sex-specific survival (**S**), and zero sub-matrices (**0**).
(TIF)

Figure S5 Seasonal harvest matrices. Each seasonal harvest matrix accounts for four disease stages for each sex and is composed of three sub-matrix elements. Panels A and B contain the summer H^(s)^ and winter H^(w)^ harvest matrices, respectively and are composed of the following sub-matrix elements: sex-specific (indexed f or m) harvest (**H**), identity (**I**), and zero sub-matrices (**0**).
(TIF)

Methods S1 Additional details regarding the harvest data utilized and methodology provided in distinct sections including *Deer Demography and Harvest*, *Disease Stages and Transition Probabilities*, and *Model Structure*. Also included are the harvest data and demographic parameter estimates used in this study.
(DOCX)
