# Supplementary figures and images for: Transmission of Chronic Wasting Disease in Wisconsin White-Tailed Deer: Implications for Disease Spread and Management
Source: PLoS One. 2014 Mar 21;9(3):e91043. doi: 10.1371/journal.pone.0091043 (PMC3962341; doi:10.1371/journal.pone.0091043)

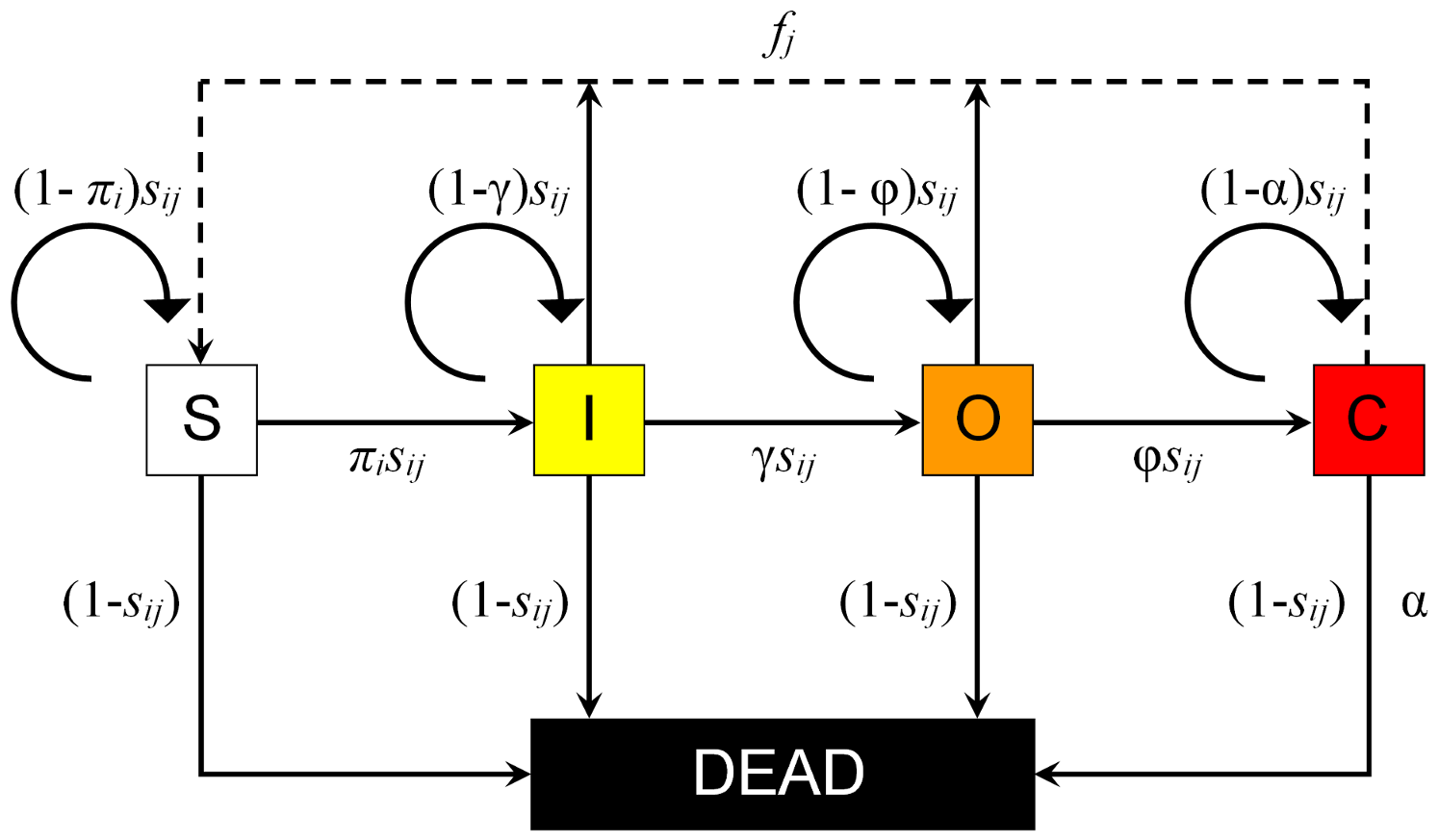

Supplement: Figure S1 — Compartmental model structure of the CWD study system. CWD stages are based on disease progression using 0.5 year time-steps. All individuals are assumed to be born susceptible (S). Infection is first detectable in retropharyngeal lymph-nodes when animals are assumed to be infectious (I). The sex-specific S-to-I transition probability is πi. Infection of the brain stem is the next detectable infection stage (O) and usually takes up to 6 months after prion detection in the alimentary lymph-nodes. The I-to-O transition probability (γ) is, hence, assumed to be equal to one. The final stage of infection is brain vacuolization which occurs 10–12 months after initial brain infection and is commonly associated with clinical signs (C). Accordingly, the O-to-C transition probability (φ) is assumed to be 0.5. From this stage most animals die within 6 months. Therefore, the disease-induced mortality probability (α) is assumed to equal one. Deer survive within and between compartments with age-sex survival probabilities sij for the j th age of sex i and reproduce with age-specific fecundity probabilities fj. (TIF) [file pone.0091043.s001.tif]

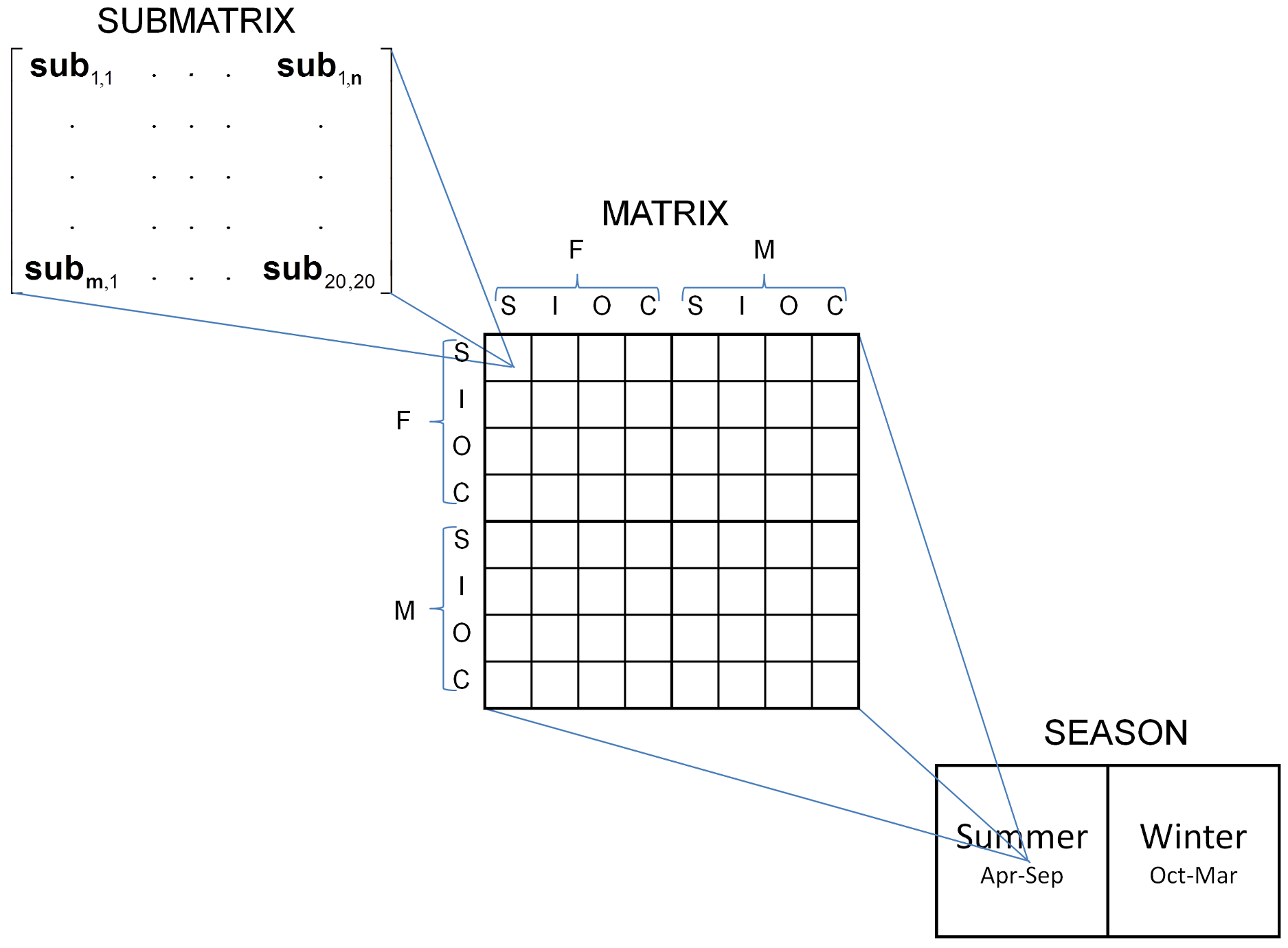

Supplement: Figure S2 — General matrix structure organization. The general hierarchical structure of the model where age-specific sub-matrices (20 6-month steps) of demographic, epidemiologic, and harvest parameters are nested within matrices accounting for four disease stages and both sexes. These matrices are further nested within season (summer and winter). Disease stages include S (Susceptible), I (Infectious), O (Obex brain positive), and C (Clinical) with F and M representing female and male deer, respectively. (TIF) [file pone.0091043.s002.tif]

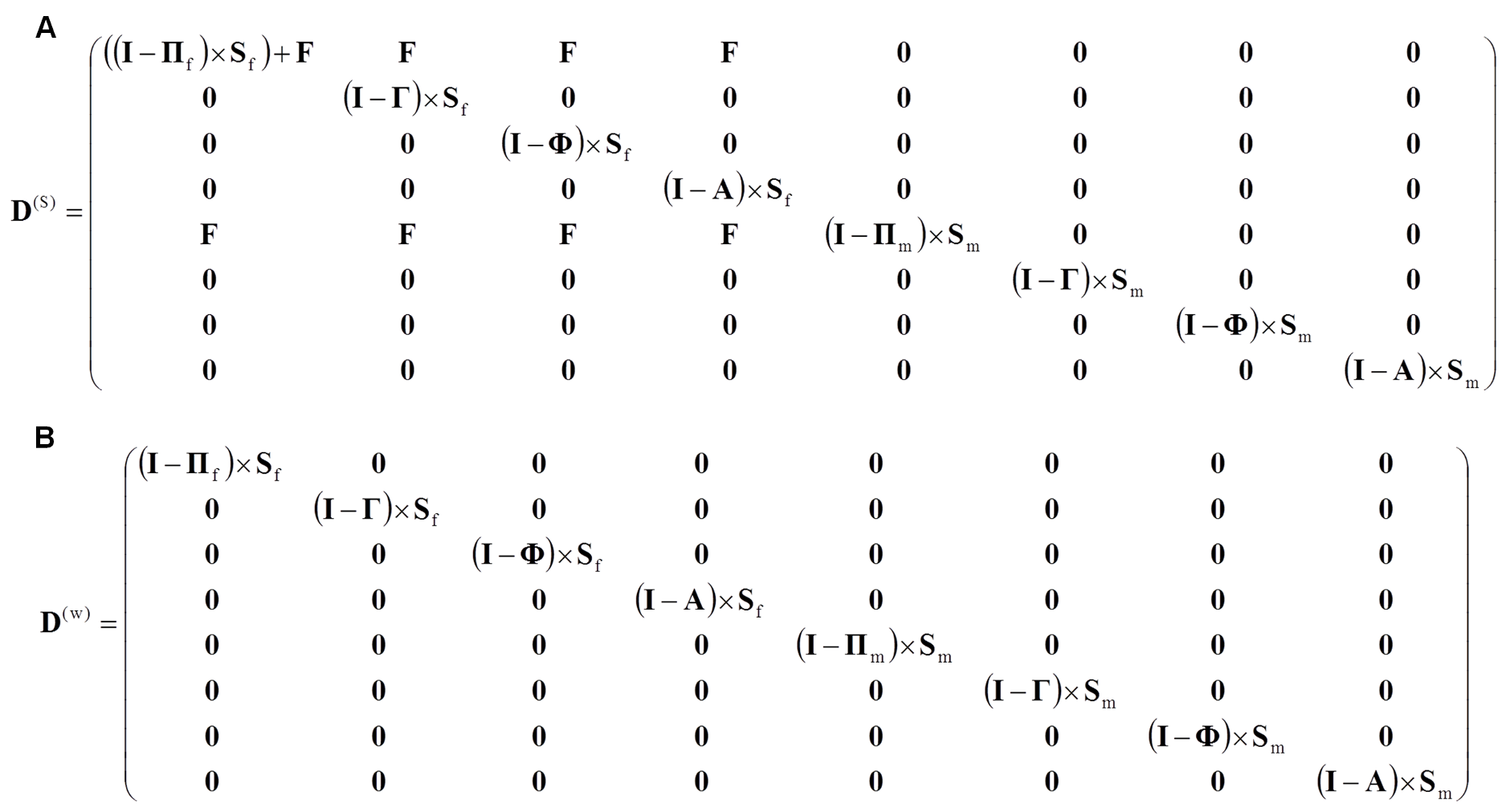

Supplement: Figure S3 — Seasonal demographic matrices. Each seasonal demographic matrix accounts for four disease stages for each sex, and is composed of eight sub-matrix elements. Panels A and B contain the summer D(s) and winter D(w) demographic matrices, respectively and are composed of the following sub-matrix elements: sex-specific (indexed f or m) transition from Susceptible to Infectious stages (Πi), transition from Infectious to Obex positive stages (Γ), transition from Obex positive to Clincial stages (Φ), transition from Clinical stage to death (A), sex-specific survival (S), fecundity (F), identity (I), and zero sub-matrices (0). (TIF) [file pone.0091043.s003.tif]

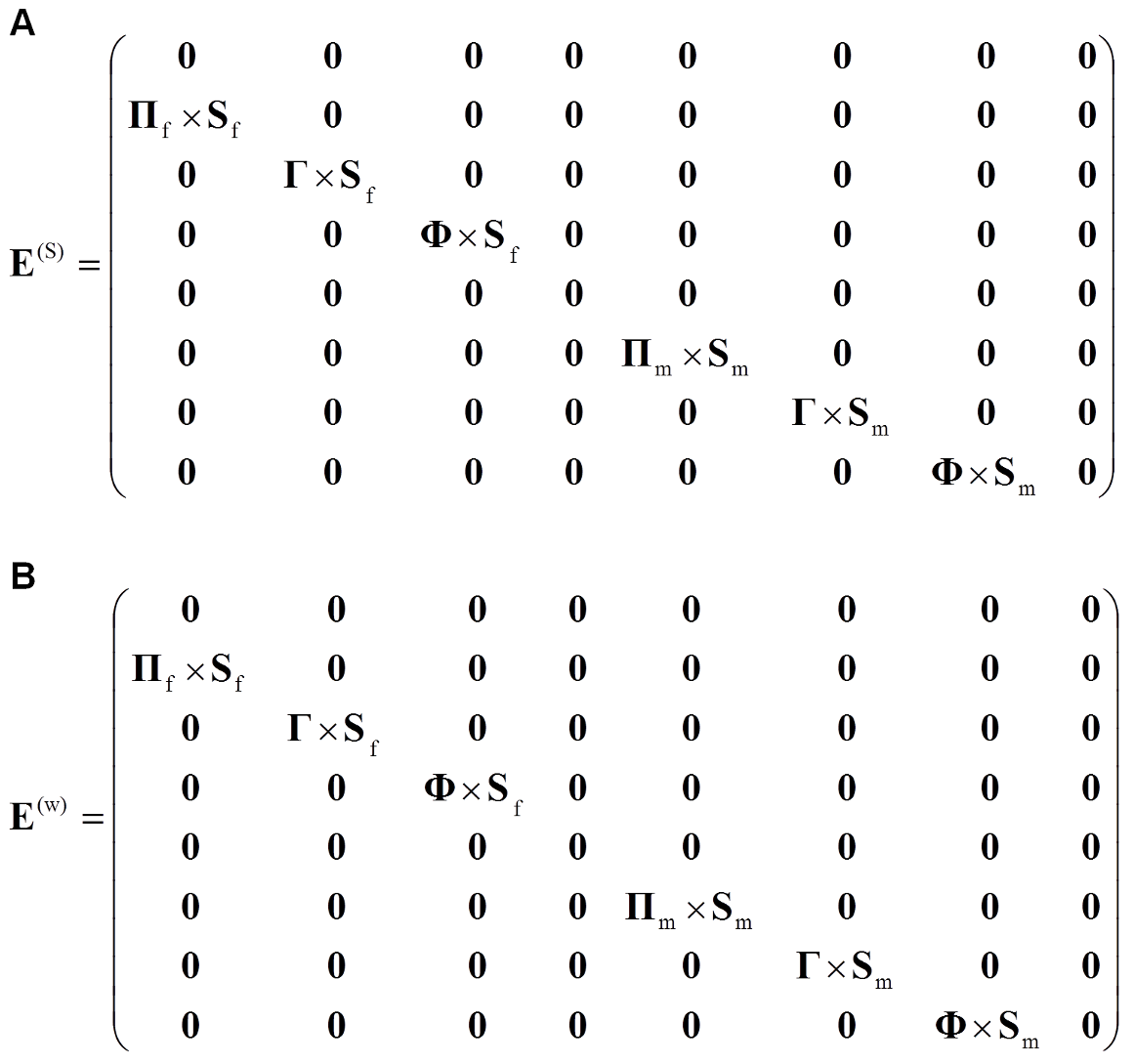

Supplement: Figure S4 — Seasonal epidemiological matrices. Each seasonal epidemiological matrix accounts for four disease stages for each sex and is composed of five sub-matrix elements. Panels A and B contain the summer E(s) and winter E(w) epidemiological matrices, respectively and are composed of the following sub-matrix elements: sex-specific (indexed f or m) transition from Susceptible to Infectious stages (Πi), transition from Infectious to Obex positive stages (Γ), transition from Obex positive to Clincial stages (Φ), sex-specific survival (S), and zero sub-matrices (0). (TIF) [file pone.0091043.s004.tif]

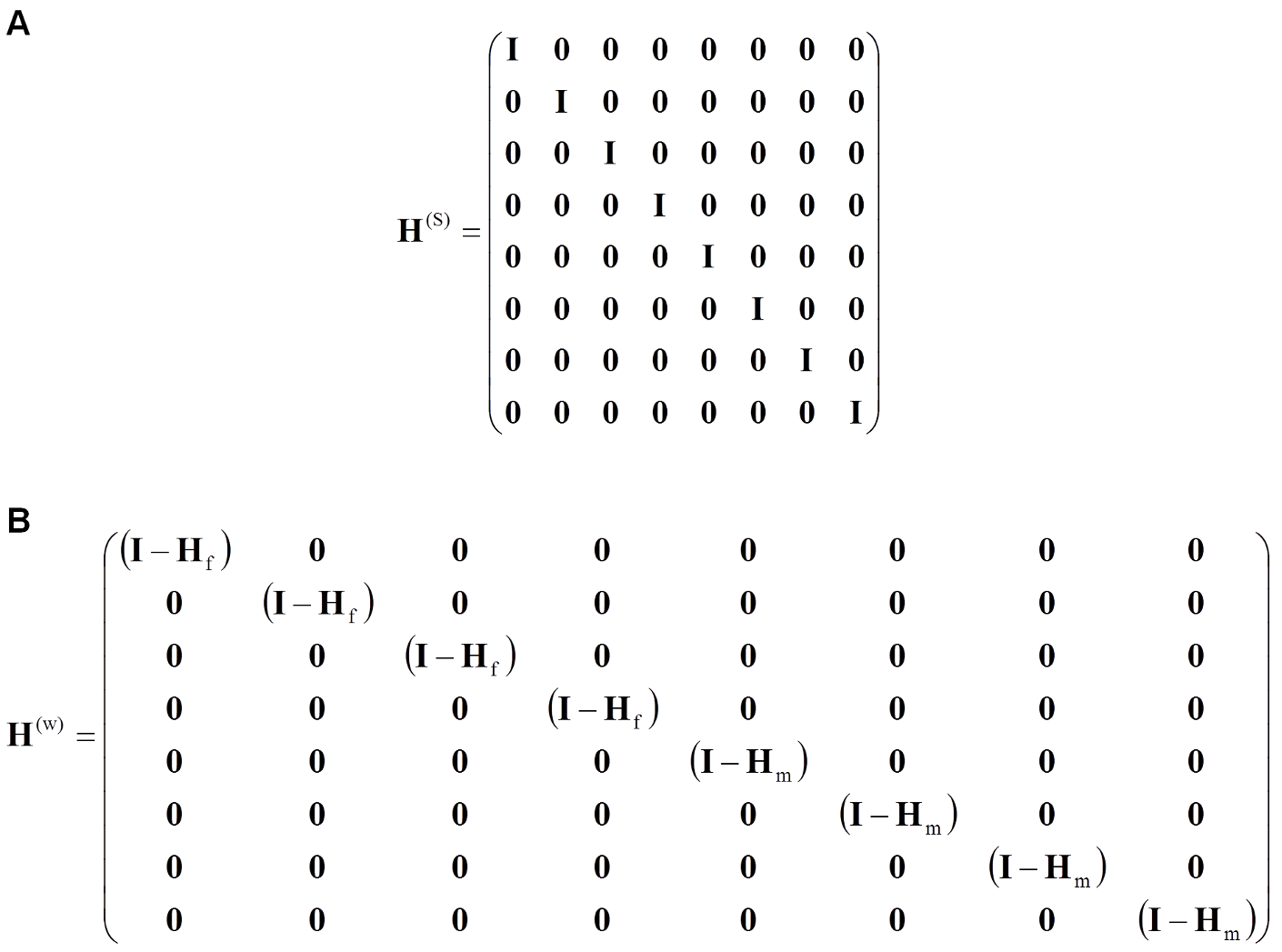

Supplement: Figure S5 — Seasonal harvest matrices. Each seasonal harvest matrix accounts for four disease stages for each sex and is composed of three sub-matrix elements. Panels A and B contain the summer H(s) and winter H(w) harvest matrices, respectively and are composed of the following sub-matrix elements: sex-specific (indexed f or m) harvest (H), identity (I), and zero sub-matrices (0). (TIF) [file pone.0091043.s005.tif]
