# Supplementary material for: Transmission of Chronic Wasting Disease in Wisconsin White-Tailed Deer: Implications for Disease Spread and Management
Source: PLoS One. 2014 Mar 21;9(3):e91043. doi: 10.1371/journal.pone.0091043 (PMC3962341; doi:10.1371/journal.pone.0091043)
Supplement: Methods S1 — Additional details regarding the harvest data utilized and methodology provided in distinct sections including Deer Demography and Harvest, Disease Stages and Transition Probabilities, and Model Structure. Also included are the harvest data and demographic parameter estimates used in this study. (DOCX) [file pone.0091043.s006.docx]

**Methods S1.**

DEER DEMOGRAPHY AND HARVEST

Our model used semi-annual time-steps to accommodate parturition and fawn rearing during summer (Apr – Sep), hunting and increased natural mortality during winter (Oct – Mar) (Table S2), and the slow rate of CWD progression for infected deer [1]. The model is non-spatial and assumes a deer population with no immigration or emigration. Deer life-span in southern Wisconsin is approximately 10 years [2], thus, we divided the deer population into 20 semi-annual age groups. Fecundity (assuming equal sex ratio at birth) was estimated as a product of the pregnancy rate and number of fawns produced by pregnant females in each age class [3] (Table S2). Non-hunting survival rates were provided by Wisconsin Department of Natural Resources (WDNR) deer ecologists (Table S2).

Deer hunting is the principal population and disease control measure used to manage deer abundance [2,4]. Throughout the paper, we generally used ‘harvest strategy’ to indicate population reduction achieved by changing the recreational harvest rates of deer. Deer hunting occurs in Wisconsin during the late fall and within the core area continues into the winter [5] (i.e. within the ‘Winter’ season of the model) and harvest is independent of CWD infection stage [6]. We used antlered (bucks ≥1 year old) and antlerless (fawns and females) harvest rates based on sex, age, kill (SAK) statistics for antlered and antlerless deer and additional deer demographic data collected by WDNR. The average annual harvest rate prior to the detection of CWD (harvest strategy) was estimated as 48% and 26% for antlered and antlerless deer, respectively. These rates produced an approximately stable deer population (*λ* ≈ 1.0) from 1987 to 2001 [1].

To estimate *TDI* and specific transmission coefficients, we estimated the deer population in the core area prior to 2001. To do that we used the Iowa County deer harvest registration, which dates back to the 1940s, but has some gaps in early years, and is continuous since 1953. Iowa County comprises more than half of south-central Wisconsin’s CWD core area and adjacent counties have less data available. Prior to and including the 1940’s, white-tailed deer were rare (< 0.25 animal km^-2^). Prior to discovery of CWD in Wisconsin in the 2001 harvest season, deer density in the study area was estimated at ≈ 9.3 deer km^-2^ based on harvest registration records. We simulated deer population size (*N*) in year *t* from 1945 to 2001 using a phenomenological logistic growth model with three parameters:

$N_{t}=\frac{N_{tol}}{1+\left[\left(N_{tol}-N_{0}\right)/N_{0}\right]e^{-rt}}$,

defined by intrinsic rate of growth (*r* = 0.297), tolerance capacity (*N*_tol_ = 5040), and starting population size (*N*_0_ = 2). The starting population size was < 0.004 deer km^-2^ (or < 0.01 deer mi^-2^), and we considered a range of sizes from 2 to 544 deer (1 deer km^-2^). We found that the starting population size did not affect model fit to the data, and infection coefficients and *TDI* varied < 5% across different starting population sizes. *N*_tol_ represents the regulatory effect of societal tolerance for deer, thus *N*_tol_ = 9.3 deer km^-2^ (9.3 ∙ 544 km^-2^ ≈ 5040 deer) is an asymptotic population tolerance density that harvest registration data suggests was maintained in the study area prior to CWD discovery.

Following the detection of CWD in 2001, liberal regulations extended the hunting period and encouraged harvest of antlerless deer with the stated goal to reduce transmission via population reduction [4]. We simulated/projected future deer population dynamics under four harvest strategies: (1) no harvest, (2) male-focused (*h*_f_ **=** 25%, *h*_m_ = 50%), (3) herd-control (*h*_f_ = 28%, *h*_m_ = 22%), and (4) female-focused *h*_f_ **=** 50%, *h*_m_ = 25%). The herd-control strategy is based on the estimated average rate of harvest during 2002-2010, during which there were changing harvest policies. For each harvest strategy we assumed no resource limitation on deer abundance [2] and density-dependent harvest at year *t*, strategy *s*, and sex *i* using a post-harvest societal tolerance level (*N*_tol_) in the core area of 5,040 deer. Thus, we defined density-dependent harvest rates by *RH_is_*_(_*_t_*_)_ = (*N_is_*_(_*_t_*_)_ / *N*_tol_) ∙ *h_is_*_(_*_t_*_)_, where *h_is_*_(_*_t_*_)_ is the nominal harvest rate with imposed constraints on RH, such that 10% ≤ *RH_is_* ≤ 50%. Because our model was deterministic, disease extinction was defined to occur when the number of infected deer becomes less than unity.

DISEASE STAGES AND TRANSITION PROBABILITIES

CWD is considered a chronic disease, with disease progression and incubation requiring 15 – 25 months from oral infection to clinical stages in captive mule deer [7,8]. We used four compartments to depict discrete stages of CWD progression (Fig. S1). Disease state progression was based on captive infection studies [7], and because there is no evidence of vertical transmission, individuals are assumed to be born susceptible (S) [9]. Infection probability (*π_i_*) determines when retropharyngeal (and other) lymph-nodes typically become disease positive (early in disease progression; 1.5 – 3 month post infection) at which time animals are believed to be infectious (I) [10–12]. For simplicity and due to lack of scientific information, we assumed that infectiousness is equal among disease stages. Infection of the brain stem or obex is typically the next stage (O) and takes < 6 months after prion detection in the alimentary lymph-nodes with transition probability γ = 1 [8]. Clinical CWD signs (C) result from vacuolization of brain tissues and occur 10–12 months after initial brain infection with corresponding transition probability φ = 0.5. Because CWD is a uniformly fatal disease we assumed clinical stage animals die within 6 months, with transition probability α = 1. Thus, the mean time from infection (i.e. entering stage I) to disease induced death is ≈ 24 months. From a previous analysis [1], force-of-infection, host population growth rate, and equilibrium prevalence were not sensitive to these disease progression parameters.

The transmission probability (*π_i_*), is the discrete-time per-capita probability of becoming infected and infectious (I) within a given time period (incidence rate). We calculated *π_i_* = 1-exp(-*λ_i_* ∆t) with *λ_i_* the force-of-infection for sex *i* and ∆t = 0.5 year [13]. We estimated the mean transmission probability across all ages for each sex *i*. Assuming homogeneous contacts among and between each sex, we modeled *λ_i_* as a linear function of the number of infectious individuals in the population by *λ_i_* = *β_i_* ∙ I for DD-transmission [14] and *λ_i_* = *β′_i_* ∙ (I ∕N) for FD-transmission [15] where *β_i_* or *β_i_′* is the transmission coefficient (i.e. the per-capita instantaneous rate of infectious contacts), (I) are the number of infected individuals, and (N) is the population size. For our non-linear model, *λ_i_* = (*β_i_* ∙ I) ∕ (1−*ε_i_* + (*ε_i_* ∙ N)) with scaling coefficient (*ε*), which ranges from 0 to 1 [16]. As *ε*→0, *λ_i_* = *β_i_* ∙ I and as *ε*→1, *λ_i_* = *β′_i_* ∙ (I ∕N). Note that *β_i_* and *β_i_′* have different dimensions [16].

In our matrix model, *π_i_* is updated at every semi-annual time-step based on the number of infected deer (DD transmission) or disease prevalence (FD transmission) in the population. The transmission coefficients (*β_i_* and *β_i_′*) we report represent annual time steps. We used a maximum-likelihood profile approach [17] to obtain parameter estimates and variance of transmission coefficients (*β_i_* or *β_i_′*) and time-since-disease-introduction (*TDI*). We used a binomial likelihood to find the maximum-likelihood estimate of the parameters *β_i_* (or *β′_i_*) and *TDI* (time since disease introduction) given the sex-age prevalence distribution of hunter-harvested deer in the southwestern CWD core area of WI from 2002-2010, and the estimated stable-age population vector in the year of CWD introduction. The form of this likelihood function was

$\text{L}(\text{β}_{\text{i}},\text{TDI}|\text{n}_{ij(t)},\text{y}_{ij(t)},\text{N}_{TDI})=\left(\begin{matrix} \text{n}_{ij(t)} \\ \text{y}_{ij(t)} \end{matrix}\right)\text{p}_{_{ij(t)}}^{\text{y}_{ij(t)}}(1-\text{p}_{_{ij(t)}})^{\text{n}_{ij(t)}-\text{y}_{ij(t)}}|\text{N}_{TDI}$, where *n_ij_*_(_*_t_*_)_ is the sample size of all hunter-harvested deer tested for CWD in year *t* (2002-2010), age class *j* (fawns, yearlings, 2, 3, and 4+ year olds), and sex *i*, *y_ij_*_(_*_t_*_)_ is the number of hunter-harvested deer that tested positive for CWD in year *t*, age class *j*, and sex *i*, and *p_ij_*_(_*_t_*_)_ is the model-predicted probability (given *β_i_* and *TDI*) that hunter-harvested deer in year *t*, age class *j*, and sex *i* were CWD positive. *N*_TDI_ is the simulated deer population vector distributed with stable age distribution in that year (*TDI* < 2002) which corresponds with the estimated year of introduction of an index CWD infected (stage I) 2-year-old female. We estimated *TDI* between 1945 and 2001, by introducing a single 2-year-old CWD-infected female into the simulated deer population (*N*_TDI_). *TDI* is derived by subtracting 2002 (discovery of CWD in WI and first year of observed data) from the CWD introduction year (subject to the simulated population size) that results in maximizing *L*(*β_i_*, *TDI*). Note that *N*_TDI_ was estimated using a phenomenological logistic population model (discussed above) based on harvest records from Iowa County. The deer population in each year grew according to demographic, epidemiologic, and harvest parameters presented in Table S2, and the Likelihood was evaluated by maximizing the fit of the observed sex-age prevalence data to model-predicted prevalence (given *β_i_* or *β′_i_* and *TDI*).

MODEL STRUCTURE

In our multistate model there are two seasons per year over which demographic, epidemiologic, and harvest dynamics proceed; summer (Apr-Sep) and winter (Oct-Mar). Each season-specific demographic, epidemiologic, and harvest matrix is composed of embedded sub-matrix elements (Fig.S2). The model incorporates four infection stages and 20 6-month increment age-classes for each sex, resulting in a transition matrix of 160×160 cells. Fawns enter the population in the summer time-step, survive and age for each semi-annual time-step or die, and conditional on survival will transition between disease stages based on assigned transition parameters.

We implemented the model in MATLAB (Mathworks, version 7.1) using sub-matrices (of dimension *r* = 20 × *c* = 20) for survival (**S*_i_***), fecundity (**F**), infection stages (**П*_i_***, **Г**, **Ф**, and **А**), and harvest (**H*_i_***) for sex *i*. We also utilized identity (**I**) and zero (**0**) structural sub-matrices. The survival sub-matrix is parameterized along the sub-diagonal where *s_rc_* for *r* ≠ 1 is the probability of surviving from age *c* to age *r* (0.5 year increments). The fecundity sub-matrix is parameterized for each age-specific element along *r* = 1 and *c* ≠ 1,2. The infection stage matrices are parameterized along the diagonal with transition probabilities *π_i_*, γ, φ, and α, respectively. The harvest sub-matrix is parameterized along the diagonal for sex-specific harvest probability in year *t*.

All sub-matrices are embedded within sex-infection stage matrices (of dimension *r* = 8 × *c* = 8) (Fig.S2). The seasonal demographic transitions are dictated by the probability of individuals remaining in the S, I, O or C infection stages, given by the sub-matrix expressions: (**I**-**П*_i_***)∙**S*_i_***, (**I**-**Г**)∙**S*_i_***, (**I**-**Ф**)∙**S*_i_***, and (**I**-**А**)∙**S*_i_*** (Fig.S3). These demographic matrices are divided into summer **D^(s^**^)^ when fecundity is nonzero and winter **D^(w^**^)^ when fecundity is zero. Seasonal epidemiological transitions between infection stages occur along the sub-diagonal of **E^(s^**^)^ and **E^(w^**^)^ according to the sub-matrix expressions **П*_i_*S*_i_*** (S-to-I), **ГS*_i_*** (I-to-O)**,** and **ФS*_i_*** (O-to-C) (Fig. S4). Harvest matrices are also subdivided by season and are parameterized to express the proportion of individuals remaining (due to harvest) in each sex-infection stage class (Fig.S5). There is no harvest in summer **H^(s^**^)^, thus the proportion of individuals surviving from harvest effort in each stage is unity (expressed by identity sub-matrices along the diagonal). In winter **H^(w^**^)^, there is sex-specific harvest expressed in the matrix as the proportion of individuals in each sex-infection stage surviving from harvest effort. Note that what we term females encompasses all antlerless deer, which includes females and fawns of both sexes.

We combined the demographic, epidemiologic, and harvest matrix components for summer (f > 0 and h = 0 ) and winter (f = 0 and h > 0) separately according to the functions **M^(s)^** = (**D^(s)^** + **E^(s)^**) ∙ **H^(s)^** and **M^(w)^** = (**D^(w)^** + **E^(w)^**) ∙ **H^(w)^**, respectively. Let **n***_t_* = $\left[ n_{1},n_{2}, \ldots,n_{160} \right]_{t}^{\text{T}}$ , the column vector of sex-infection stage-age abundance at time *t*, with T representing the transpose operator. Thus, the abundance of each sex-infection stage-age class in time *t*+1 is:

**n***_t_*_+1_ = (**M^(w)^** ∙ **M^(s)^**) **n***_t_*

From the product of **M^(w)^** ∙ **M^(s)^** we obtained the eigenvector corresponding to the dominant eigenvalue, which represents the stable age distribution.

REFERENCES

1. Wasserberg G, Osnas EE, Rolley RE, Samuel MD (2009) Host culling as an adaptive management tool for chronic wasting disease in white-tailed deer: a modelling study. J Appl Ecol 46: 457–466. doi:10.1111/j.1365-2664.2008.01576.x.

2. Wisconsin Department of Natural Resources (2001) Management workbook for white-tailed deer. Second edition. Madison, WI, USA: Bureaus of Wildlife Management and Integrated Science Services.

3. McCaffery KR, Ashbrenner JE, Rolley RE (1998) Deer Reproduction in Wisconsin. Trans Wis Acad Sci Arts Lett 86: 249–262.

4. Bartelt GA, Pardee J, Thiede K (2003) Environmental impact statement on rules to eradicate chronic wasting disease in Wisconsin’s free-ranging white-tailed deer herd. Wisconsin Department of Natural Resources, Bureau of Integrated Science Services. 175 p.

5. Rolley RE (2005) Controlling chronic wasting disease in Wisconsin : a progress report and look toward the future. Madison, Wisconsin: Wisconsin Department of Natural Resources, Bureaus of Wildlife Management and Integrated Science Services. 20 p.

6. Grear DA, Samuel MD, Langenberg JA, Keane D (2006) Demographic Patterns and Harvest Vulnerability of Chronic Wasting Disease Infected White-Tailed Deer in Wisconsin. J Wildl Manag 70: 546–553. doi:10.2193/0022-541X(2006)70[546:DPAHVO]2.0.CO;2.

7. Sigurdson CJ, Williams ES, Miller MW, Spraker TR, O’Rourke KI, et al. (1999) Oral transmission and early lymphoid tropism of chronic wasting disease PrPres in mule deer fawns (Odocoileus hemionus). J Gen Virol 80 ( Pt 10): 2757–2764.

8. Williams ES, Miller MW (2002) Chronic wasting disease in deer and elk in North America. Rev Sci Tech Int Off Epizoot 21: 305–316.

9. Miller MW, Williams ES (2003) Prion disease: Horizontal prion transmission in mule deer. Nature 425: 35–36. doi:10.1038/425035a.

10. Hagenaars TJ, Donnelly CA, Ferguson NM, Anderson RM (2003) Dynamics of a scrapie outbreak in a flock of Romanov sheep – estimation of transmission parameters. Epidemiol Infect 131: 1015–1022. doi:10.1017/S0950268803001055.

11. Miller MW, Wild MA (2004) Epidemiology of chronic wasting disease in captive white-tailed and mule deer. J Wildl Dis 40: 320–327.

12. Miller MW, Hobbs NT, Tavener SJ (2006) Dynamics of prion disease transmission in mule deer. Ecol Appl 16: 2208–2214.

13. Caley P, Ramsey D (2001) Estimating disease transmission in wildlife, with emphasis on leptospirosis and bovine tuberculosis in possums, and effects of fertility control. J Appl Ecol 38: 1362–1370. doi:10.1046/j.0021-8901.2001.00676.x.

14. Anderson RM, May RM (1991) Infectious diseases of humans: dynamics and control. Oxford; New York: Oxford University Press. 757 p.

15. Rudolf VHW, Antonovics J (2005) Species coexistence and pathogens with frequency-dependent transmission. Am Nat 166: 112–118. doi:10.1086/430674.

16. McCallum H, Barlow N, Hone J (2001) How should pathogen transmission be modelled? Trends Ecol Evol 16: 295–300.

17. Hilborn R, Mangel M (1997) The Ecological Detective: Confronting Models with Data. Princeton: Princeton University Press. 330 p.

**Table S1. Numbers of deer tested for CWD in the southwestern core area of Wisconsin from the 2002 to 2012 harvest seasons distributed by sex (F=female, M=male) and age (fawns, 1=yearlings, 2=2-year-olds, 3=3-year-olds, and 4+ = 4-year-olds and older).**

| Uninfected | 2002 | 2003 | 2004 | 2005 | 2006 | 2007 | 2008 | 2009 | 2010 | 2011 | 2012 |
| --- | --- | --- | --- | --- | --- | --- | --- | --- | --- | --- | --- |
| F fawns | 786 | 153 | 149 | 97 | 74 | 58 | 52 | 40 | 43 | 12 | 13 |
| F 1 | 470 | 264 | 231 | 170 | 124 | 215 | 163 | 144 | 110 | 80 | 74 |
| F 2 | 387 | 214 | 272 | 175 | 136 | 203 | 183 | 130 | 146 | 83 | 68 |
| F 3 | 253 | 124 | 142 | 101 | 84 | 121 | 165 | 112 | 89 | 57 | 48 |
| F 4+ | 263 | 89 | 125 | 82 | 72 | 102 | 87 | 75 | 108 | 54 | 47 |
| M fawns | 772 | 172 | 142 | 111 | 66 | 46 | 37 | 37 | 47 | 27 | 13 |
| M 1 | 542 | 388 | 259 | 260 | 337 | 245 | 140 | 146 | 83 | 181 | 161 |
| M 2 | 361 | 250 | 267 | 265 | 253 | 171 | 129 | 114 | 89 | 135 | 107 |
| M 3 | 178 | 122 | 145 | 142 | 115 | 98 | 103 | 104 | 90 | 126 | 97 |
| M 4+ | 21 | 16 | 16 | 10 | 27 | 15 | 17 | 16 | 23 | 36 | 26 |
| Infected |  |  |  |  |  |  |  |  |  |  |  |
| F fawns | 2 | 0 | 1 | 0 | 0 | 1 | 0 | 0 | 2 | 0 | 0 |
| F 1 | 10 | 5 | 4 | 3 | 3 | 7 | 9 | 10 | 2 | 5 | 2 |
| F 2 | 19 | 8 | 8 | 9 | 6 | 7 | 12 | 10 | 10 | 3 | 7 |
| F 3 | 15 | 5 | 7 | 3 | 3 | 7 | 8 | 9 | 11 | 7 | 9 |
| F 4+ | 11 | 4 | 5 | 7 | 6 | 6 | 9 | 4 | 8 | 5 | 4 |
| M fawns | 2 | 0 | 0 | 1 | 0 | 0 | 0 | 0 | 0 | 0 | 0 |
| M 1 | 14 | 13 | 5 | 7 | 5 | 7 | 9 | 6 | 8 | 10 | 11 |
| M 2 | 32 | 24 | 33 | 27 | 20 | 16 | 24 | 19 | 24 | 20 | 23 |
| M 3 | 29 | 11 | 13 | 19 | 24 | 13 | 20 | 11 | 28 | 35 | 29 |
| M 4+ | 3 | 2 | 3 | 3 | 2 | 3 | 0 | 1 | 9 | 11 | 11 |

**Table S2. White-tailed deer demographic and harvest parameters.** Fecundity rate (*f*) is based on [3]. Survival rate (*S*) is the age-specific non-harvest survival probability (WDNR, unpublished data). Harvest rate (*h*) is mean harvest rate for antlered and antlerless deer for the years 1987 – 2001 (prior to detection of CWD).

| Age | Season | Males | | Female | | |
| --- | --- | --- | --- | --- | --- | --- |
|  |  | *S* | *h* | *f* | *S* | *h* |
| Fawn | Summer | 0.92 | - | - | 0.92 | - |
|  | Winter | 0.95 | 0.26 | - | 0.95 | 0.26 |
| Yearling | Summer | 0.97 | - | 0.293 | 0.97 | - |
|  | Winter | 0.97 | 0.48 | - | 0.97 | 0.26 |
| 2 | Summer | 0.96 | - | 0.730 | 0.97 | - |
|  | Winter | 0.96 | 0.48 | - | 0.97 | 0.26 |
| 3+ | Summer | 0.96 | - | 0.903 | 0.97 | - |
|  | Winter | 0.96 | 0.48 | - | 0.97 | 0.26 |
